# The wild grape genome sequence provides insights into the transition from dioecy to hermaphroditism during grape domestication

**DOI:** 10.1186/s13059-020-02131-y

**Published:** 2020-09-07

**Authors:** Hélène Badouin, Amandine Velt, François Gindraud, Timothée Flutre, Vincent Dumas, Sonia Vautrin, William Marande, Jonathan Corbi, Erika Sallet, Jérémy Ganofsky, Sylvain Santoni, Dominique Guyot, Eugenia Ricciardelli, Kristen Jepsen, Jos Käfer, Hélène Berges, Eric Duchêne, Franck Picard, Philippe Hugueney, Raquel Tavares, Roberto Bacilieri, Camille Rustenholz, Gabriel A. B. Marais

**Affiliations:** 1 Université de Lyon, Université Lyon 1, CNRS, Laboratoire de Biométrie et Biologie Evolutive UMR 5558, F-69622 Villeurbanne, France; 2 Université de Strasbourg, INRAE, SVQV UMR-A 1131, F-68000 Colmar, France; 3 GQE–Le Moulon, INRAE, Univ. Paris-Sud, CNRS, AgroParisTech, Univ. Paris-Saclay, 91190 Gif-sur-Yvette, France; 4 INRAE, Centre National de Ressources Génomiques Végétales, F-31326 Castanet-Tolosan, France; 5 LIPM, Université de Toulouse, INRAE, CNRS, Castanet-Tolosan, France; 6 INRAE, UMR AGAP, Univ. Montpellier, CIRAD, INRAE, Institut Agro, Montpellier, France; 7 PRABI, Université Lyon 1, F-69622 Villeurbanne, France; 8 IGM Genomics Center, University of California, San Diego, La Jolla, CA USA

**Keywords:** Grapevine, Dioecy, Sex chromosomes, Sex-determining genes

## Abstract

**Background:**

A key step in domestication of the grapevine was the transition from separate sexes (dioecy) in wild *Vitis vinifera ssp. sylvestris* (*V. sylvestris*) to hermaphroditism in cultivated *Vitis vinifera ssp. sativa* (*V. vinifera*). It is known that *V. sylvestris* has an XY system and *V. vinifera* a modified Y haplotype (Yh) and that the sex locus is small, but it has not previously been precisely characterized.

**Results:**

We generate a high-quality de novo reference genome for *V. sylvestris*, onto which we map whole-genome re-sequencing data of a cross to locate the sex locus. Assembly of the full X, Y, and Yh haplotypes of *V. sylvestris* and *V. vinifera* sex locus and examining their gene content and expression profiles during flower development in wild and cultivated accessions show that truncation and deletion of tapetum and pollen development genes on the X haplotype likely causes male sterility, while the upregulation of a Y allele of a cytokinin regulator (*APRT3*) may cause female sterility. The downregulation of this cytokinin regulator in the Yh haplotype may be sufficient to trigger reversal to hermaphroditism. Molecular dating of X and Y haplotypes is consistent with the sex locus being as old as the *Vitis* genus, but the mechanism by which recombination was suppressed remains undetermined.

**Conclusions:**

We describe the genomic and evolutionary characterization of the sex locus of cultivated and wild grapevine, providing a coherent model of sex determination in the latter and for transition from dioecy to hermaphroditism during domestication.

## Background

Dioecy is rare in flowering plants (~ 6%) but over-represented among crops (~ 20%) [1]. In some cases, both wild and cultivated plants are dioecious (e.g., date palm, asparagus, persimmons). Other crops, such as grapevine, papaya, and strawberry, derive from dioecious progenitors and switched to hermaphroditism during domestication. The genes underlying this switch are currently not known in any crop. In *Vitis vinifera* ssp. *sylvestris* (*V. sylvestris*), the wild ancestor of domesticated and cultivated grapevine *Vitis vinifera* ssp. *vinifera* (*V. vinifera*), wild females produce morphologically bisexual flowers, with retracted anthers that produce few and infertile pollen, while male flowers undergo early ovule abortion [2, 3]. Old genetic studies suggested that in grape, sex is determined by one locus with three alleles M, F, and H [3]. In *V. sylvestris*, males are MF and females FF, and in *V. vinifera*, hermaphrodites can be HF or HH [2, 3]. A genetic analysis based on a population segregating for sex, obtained by crossing a *V. vinifera* genotype and an interspecific rootstock, allowed the identification of a 143-kb sex locus located on chromosome 2 in the *V. vinifera* reference genome [4]. However, this particular region is not well assembled in the reference genome (PN40024—version 12X.2) [5], the F and H haplotypes being mixed both on chr2 and on unassembled scaffold_233 [6]. Moreover, other genetic studies in *V. vinifera* found different size and boundaries for the sex locus (e.g., [7, 8]).

In *V. sylvestris*, a target sequencing in a core-collection of males and females using the genes previously identified in *V. vinifera* confirmed the sex-linkage of some of them, but not all [6]. It appeared that *V. sylvestris* has a XY system in which M = Y and F = X. It was also found that the H haplotype of *V. vinifera* is likely a modified Y haplotype (hereafter called Yh) [6]. Differential expression analysis of male, female, and bisexual flowers in *V. sylvestris* and *V. vinifera* identified two candidate genes for female sterility, in particular *APRT3*, a putative cytokinin regulator located on chromosome 2 of *V. vinifera* [9], but no causative mutations have been identified. A genetic and evolutionary model to explain sex determination and switch to hermaphroditism in grapevine is currently lacking.

To build such a model, we aimed at (i) characterizing first the sex locus in *V. sylvestris*, the wild progenitor of *V. vinifera*, and (ii) comparing the sex locus in both wild and cultivated grapevine. We first produced a reference genome of *V. sylvestris*. We then re-sequenced a *V. sylvestris* cross and used a segregation analysis to identify sex-linked SNPs and locate the sex locus on the *V. sylvestris* genome. Comparing X, Y, and Yh haplotypes from both *V. sylvestris* and *V. vinifera* obtained by BAC sequencing, we characterized the presence/absence patterns in the sex locus genes. We then compiled the transcriptomic profiles of the sex locus genes during flower development. Combining all this information, we found a few candidate sex-determining genes and identified changes between the Y and Yh haplotypes that could explain the switch from dioecy to hermaphroditism during domestication.

## Results

### Sequencing and de novo assembly of a female *V. sylvestris* genome

For this work, we first produced a PacBio-based reference genome for *V. sylvestris* that was not available yet. We chose a female individual that was sequenced using SMRT-sequencing (120X). Contigs were assembled with falcon-unzip [10], and the grapevine reference genome (PN40024—version 12X.2, [5]) was used as a framework to help building pseudomolecules. We obtained a high-quality diploid assembly of 469 Mb with a contig N50 of 1.7 Mb, 98% of the gene content anchored on chromosomes and a BUSCO evaluation of 95% (Table 1, Additional file 1: Table S1, Additional file 1: Fig. S1, Additional file 2), comparing favorably to other recently published plant genomes [11–13]. We annotated 39,031 protein-coding genes on primary contigs (Table 1).

**Table 1:** Statistics of the *V. sylvestris* genome

		Primary contigs	Anchored primary contigs	Primary pseudomolecules (without chrUn)	Primary pseudomolecules (with chrUn)	Haplotigs	Anchored haplotigs	Haplotigs pseudomolecules (without chrUn)	Haplotigs pseudomolecules (with chrUn)
**Number**		591	476	19	20	3781	3537	19	20
**Mean length (b)**		792,724	947,992	23,761,734	23,439,278	83,879	86,014	16,030,808	15,876,286
**Median length (b)**		421,056	584,495	22,587,513	22,585,150	36,553	37,333	16,051,524	15,790,240
**Max length (b)**		6,865,695	6,865,695	34,169,506	34,169,506	2,541,772	2,541,772	26,261,478	26,261,478
**L50**		82	77	8	8	483	453	8	8
**N50 (b)**		1,711,677	1,773,898	22,747,317	22,747,317	173,606	179,170	16,516,492	16,516,492
**Assembly size (b)**		468,500,071	451,244,463	451,472,963	468,785,571	317,149,633	304,233,565	304,585,365	317,525,733
**BUSCO**	**Complete and single copy**	92.6%	92.8%	93.5%	93.2%	59.7%	59.7%	61.5%	61.7%
	**Complete and duplicated**	2.6%	2.5%	1.7%	1.8%	3.8%	3.8%	1.9%	1.9%
	**Fragmented**	1.9%	1.9%	1.8%	1.9%	1.9%	1.8%	1.6%	1.7%
	**Missing**	2.9%	2.8%	3.0%	3.1%	34.6%	34.7%	35.0%	34.7%
**Number of annotated genes**		40,928	40,172	40,172	40,928	28,303	27,593	27,593	28,303
**Number of annotated protein-coding genes**		39,031	38,371	38,371	39,031	26,625	26,102	26,102	26,625

### Location and boundaries of the sex locus

On the newly obtained *V. sylvestris* assembly, the sex locus as described previously [4, 6] appeared to be fully included in the 5th largest contig of the assembly. To precisely identify the boundaries of the sex locus, a F1 family obtained from a controlled cross of *V. sylvestris* was generated and re-sequenced. Paired-end Illumina short reads of both parents and 10 offspring yielded 43 to 74 million of paired-end reads per individual (Additional file 1: Table S2). To overcome issues due to X-Y divergence, we used an iterative SNP-tolerant mapping procedure [14], eventually mapping 98.2% of reads in average (mapping coverage by individual 14x–28x, Additional file 1: Table S3–6).

**Fig. 1:**
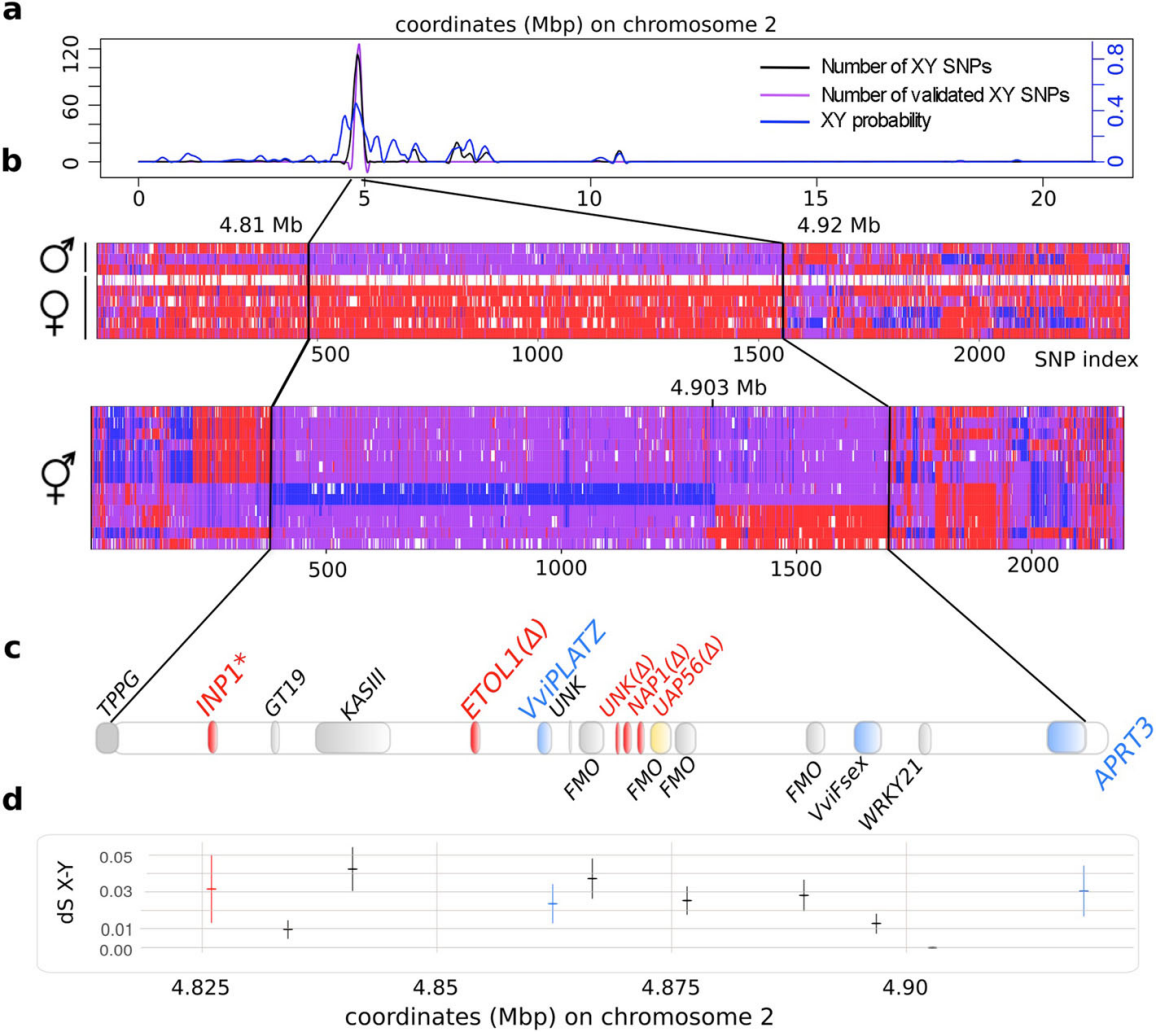
Limit, gene content and synonymous divergence in the sex locus of *V. sylvestris* and *V. vinifera*. (a) Detection of sex-linked SNPs on chromosome 2. Left *y* axis: adjusted SNP number in 10 kb windows, black curve: XY single nucleotide polymorphisms detected by SEX-DETector++ in a cross of *V. sylvestris*. Purple curve: candidate XY SNPs that show heterozygosity in males and homozygosity in females in a validation dataset of public whole-genome re-sequencing is drawn in purple; right *y*-axis (blue curve): adjusted mean posterior probability of being XY for SNPs in 10 kb windows. (b) Genotype of nine *V. sylvestris* individuals (top) and thirteen grapevine cultivars (bottom) in a validation dataset of public whole-genome re-sequencing, at locations of candidates XY SNPs. Red, purple, blue, and white marks represent XX, XY, YY, and missing genotypes, respectively. In the cultivar panel, 11 cultivars are heterozygous (XY) for the sex-linked SNPs, with a Yh haplotype closely derived from Y (see text), while two cultivars (Chardonnay and Riesling) are homozygous YhYh. YhYh genotypes were previously found only in hermaphrodite cultivars [6]. The black lines highlight the limits of shared XY SNPs between the cross re-sequenced in the present study and the validation dataset. (c) Gene content and annotation and in the sex locus (approximate position). Genes highlighted in red are absent (Δ) or possess a frameshift deletion in the X haplotype (*). Genes highlighted in blue are induced in males. The yellow gene is absent in the Y haplotype. (d) Synonymous divergence between X and Y allele in the sex locus (+/− standard error), reflecting the age of recombination suppression. dS was only computed for genes present in both haplotypes

On this data, single nucleotide polymorphisms (SNPs) that segregate with sex in our cross were identified with an empirical approach and with SEX-DETector^++^, a new version of the probabilistic method SEX-DETector that identifies sex-linked genes from patterns of segregation in a cross from RNA-seq data [15], which we further developed here to analyze DNA-seq data. As SNPs close to the sex locus might be genetically linked to the locus in a particular cross only, we used public whole-genome re-sequencing data to validate the sex-linked SNPs detected in this cross (Fig. 1a, b, Additional file 1: Tables S7–8). We searched for SNPs that were sex-linked in our cross and that were always heterozygous in males and homozygous in females in the validation dataset. We found that the X haplotype of the sex locus spanned 111 kb on the *V. sylvestris* chromosome 2 (4,810,929 to 4,921,949 bp, Fig. 1b, c, Additional file 1: Fig. S2–5), and we obtained a final dataset of 1865 XY SNPs (Additional file 3).

### Full assemblies of the X, Y, and Yh haplotypes

**Fig. 2:**
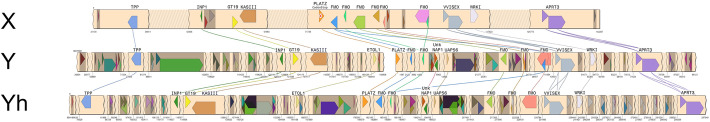
Structural variation and gene content in the three haplotypes of the sex locus. (A) X haplotype of *Vitis sylvestris*; (B) Y haplotype of *Vitis* sylvestris; (C) Yh haplotype of the Cabernet Sauvignon cultivar. The gene positions were derived from a manually curated Eugene annotation [16]. The transposable elements (TEs) were detected with RepeatMasker 4.1 [17]; only TEs longer than 300 bp are represented (grayed rectangles). The synteny among sequences was obtained with SimpleSynteny [18] and orthologous genes connected by curved lines. The graphical representation is not drawn on scale: the distance between the TPP and APRT3 genes is 1.64 and 2.12 longer for the Y and Yh haplotypes respectively, as compared to the X haplotype. Four genes (ETOL1, UAP56, NAP1, Unk) were detected only on the Y and Yh haplotypes

To explore the differences in gene content and the structural rearrangements among haplotypes of the sex locus, we sequenced and assembled bacterial artificial chromosomes (BACs) covering the sex locus in a male of another *V. sylvestris* population and in *V. vinifera* cv. Cabernet-Sauvignon. We obtained full assemblies for the X and Yh haplotypes and an assembly with a 13-kb gap in the Y haplotype located in the *VviPLATZy* gene (see “Methods” section and Additional file 1: Text S1). When aligned, the X haplotype and two BAC contigs covering the Y haplotype showed colinearity (Fig. 2, Additional file 1: Fig. S2). When including also the flanking regions in the alignment, no inversion was found (Additional file 1: Fig. S6). To identify deletions, we used the alignment between the X and the Y haplotypes (Additional file 1: Fig. S2) as well as the mean mapping coverage in our re-sequencing dataset along the X haplotype, searching for two-fold reductions in males. This revealed eight regions spanning 500 to 5500 bp and affecting one predicted gene (Additional file 1: Fig. S3). We detected more insertions of transposable elements in the Y haplotype than the X one (Table 2, Additional file 1: Fig. S2, Additional file 4, Additional file 5).

**Table 2:** Transposable element density in the X, Y and Yh haplotypes from the reference genome and the BACs

Haplotype	DNA transposons	Retro-transposons	Other repeats	Total
*V. sylvestris* X (ref. genome)	23	23	9	55
*V. sylvestris* X (BAC sequence)	26	26	10	62
*V. vinifera* cv. Cabernet Sauvignon X (BAC sequence)	25	21	9	55
*V. sylvestris* Y (BAC sequence)	41	64	6	111
*V. vinifera* cv. Cabernet Sauvignon Yh (BAC sequence)	46	89	13	148

The comparison between the X, Y, and the Yh haplotypes showed strong similarities between Y and Yh haplotypes and confirmed that the Yh haplotype derives from the Y haplotype (Fig. 2, Additional file 1: Fig. S3 and S4). We however found that transposable elements’ insertions are more numerous on the Yh haplotype compared to its Y counterpart (Table 2). Most of these insertions are located close to the *APRT3* gene (Additional file 1: Fig. S4). Our assembly of the Yh haplotype was compared with published long-read-based sequences of Yh from Zinfandel [X-Yh] [19], Cabernet Sauvignon [X-Yh] [20], and Chardonnay [Yh-Yh′] [21]. Sequence identity was respectively 99.93%, 99.97%, and 99.62%. Dot plots showed a full synteny between the Yh haplotypes. To explore the potential differences among *V. vinifera* cultivars, we investigated the SNP genotypes at the sex locus in publicly available DNA-seq data of 13 hermaphrodite grapevine cultivars [8] (Additional file 1: Table S9). Out of 13 cultivars, six harbored recombinant genotypes. The 5′ part of the sex locus was always either XYh or YhYh and spanned 93 kb (4.810–4.903 Mb) [6]. The remaining part of the sex locus (4.903–4.922 Mb) was either XX or XYh, but never YhYh (Fig. 1b, Additional file 1: Table S10). The genes essential for the male phenotype should therefore be located in the 93-kb region of the sex locus.

### Age of the haplotypes

To estimate the age of the sex locus, we calculated the synonymous divergence (dS) between X and Y coding DNA sequences (CDS) of XY gene pairs (Fig. 1c) as commonly done [22–24]. The dS values are all around 0.03 (except two genes for which dS X-Y ≤ 0.01), and the mean and median values for the sex locus are 0.024 and 0.026 respectively (Additional file 6). The homogeneity of the dS values suggests that the recombination was suppressed at about the same time for the whole locus. To obtain a rough divergence time from the dS values, we used a molecular clock (7 × 10^−9^ base substitution/site/generation time) available for plants [25]. The average generation time in years is difficult to estimate in *V. sylvestris* as individuals are sexually mature at about 5 years old in the wild but become reproductively efficient later (> 7–10 years old) and old individuals (some more than 100 years old) can be very large and productive [26]. Using a generation time of 5–10 years, we found that the sex locus could be 10.7–21.4 My old, setting a lower bound estimate. To get a higher bound estimate, we computed the average generation time from minimum and maximum age for sexual reproduction (52.5 years) and found that the sex locus could be 112.5 My old. We thus obtain a large range for the age of the sex locus, but it suggests that the sex locus is at least tens of million years old and is not recent.

We did a similar analysis for dating the Yh haplotype. As the Yh haplotype derives from the Y haplotype, we computed the dS values between Yh and Y alleles of the genes in the sex locus (Additional file 6). We found that the mean and the median dS are 0.0065 and 0 respectively, which are much lower values compared to what we obtained for the comparison between the X and Y haplotypes. Using the dS Y-Yh and using the minimum and average generation times mentioned above, we found that the Yh haplotype might be 2.32 to 24.4 MY old. Using a corrected mean (after removal of two outliers with high dS values) dS Y-Yh instead, we found that Yh haplotype might be 0.57 to 6 MY old. Our lower bound estimate is thus much higher than the estimated date of the domestication of grapevine.

### Finding candidate male-sterility genes

**Fig. 3:**
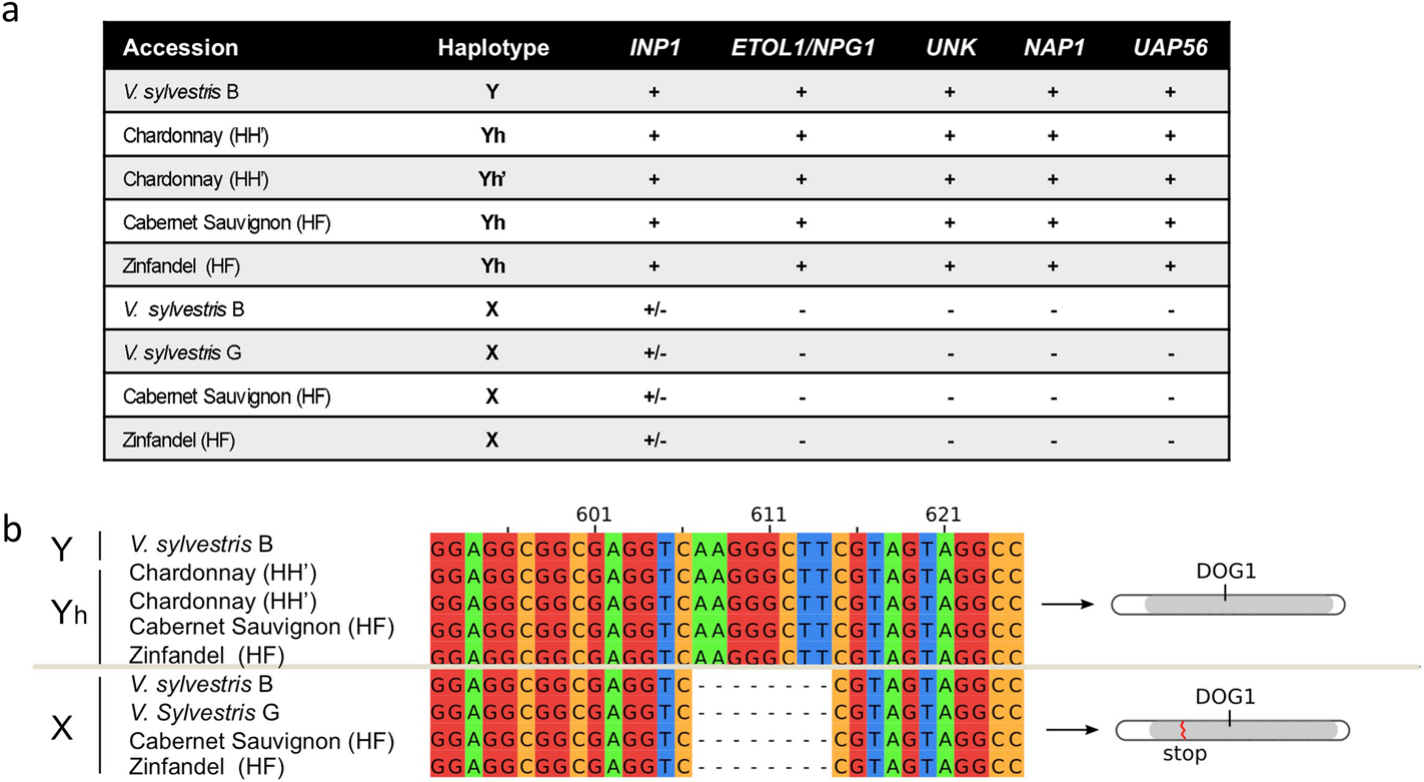
Presence-absence patterns and frameshifts mutations in X, Y, and Yh haplotypes. **a** Presence-absence patterns of five genes absent or truncated in the X haplotype of *V. sylvestris*. **b** An 8-bp deletion in exon 2 of the *INP1* gene is shared by X haplotypes and absent in Y or Yh haplotypes. It results in a premature stop codon, leading to a shorter protein. The DOG protein domain, involved in DNA binding, is truncated

**Fig. 4:**
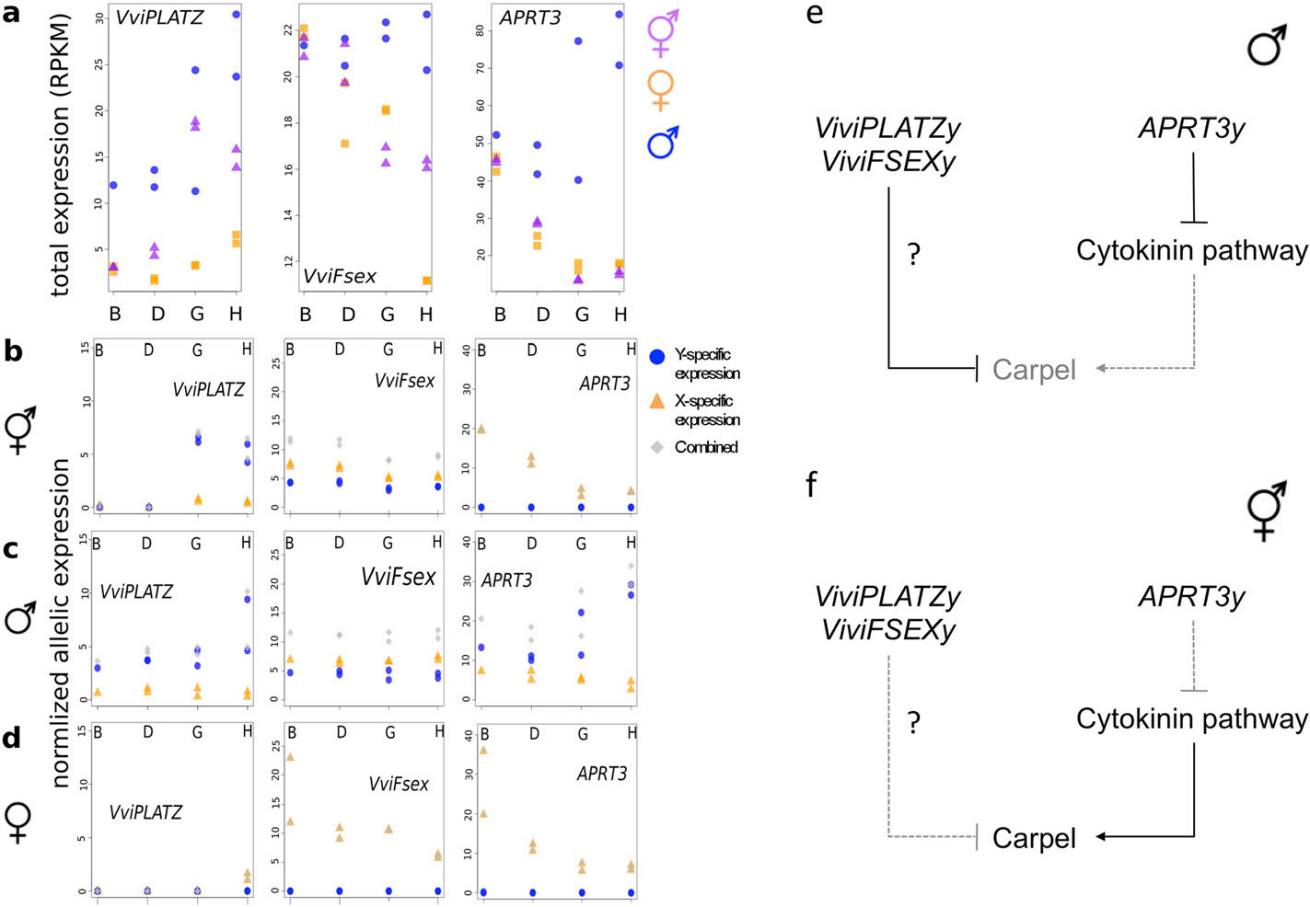
Total and allele-specific expression of female-sterility candidates during flower bud development. B, D, G, and H represent early development stages of flower development sequenced in RNA-seq in [27]. **a** Total normalized expression (RPKM) of *VviPLATZ*, *VviFsex*, and *APRT3* in males (blue circles) and females (orange squares) of *V. sylvestris*, and hermaphrodites (purple triangles) of *V. vinifera*. Each point represents a biological replicate. **b**–**d** Allele-specific normalized expression. Blue dots, orange triangles, and gray diamonds represent Y-specific, X-specific, and summed expression, respectively in the three sexes: hermaphrodite (**b**), male (**c**), and female (**d**). RNA-seq data were genotyped, and the coverage of X and Y variants was extracted from vcf file and averaged by gene. Only variants common to Y and Yh were included in the analysis of hermaphrodite data. Read counts were normalized by library. Each point represents a biological replicate. **e** Genetic model for the female-sterility mutation in *V. sylvestris*. *APRT3y* inhibits the cytokinin pathway, which results in carpel inhibition and ovule abortion. This may be reinforced by the action of *VviPLATZy* and *VviFSEXy* by unknown mechanisms. **f** Modified model in *V. vinifera*. *APRT3y* expression is not enough to trigger the carpel inhibition and ovule abortion. Actions of *VviPLATZy* and *VviFSEXy* may also be modified

The classical genetic models for sex determination in grapevine suggested that the sex locus contains two genes (with a dominant *So* = suppressing ovule allele for the first gene, and a recessive *sp* = suppressing pollen for the second one, see [3]). According to these models, females are *so sp/so sp* and males *So Sp/so sp*; the X haplotype should thus bear the *sp* recessive allele and the Y haplotype the *So* dominant one. To identify candidates, we compared the gene content of X, Y, and Yh haplotypes (Fig. 3a) and searched for presence/absence patterns and loss-of-function mutations (Fig. 3b). We also mapped a public RNA-seq dataset of males, females, and hermaphrodites flower buds of *V. sylvestris* and *V. vinifera* at four developmental stages [27] (Additional file 1: Table S11), measuring the total (Fig. 4a) and the allele-specific (X, Y, and Yh, Fig. 4b) expression of transcripts.

First, we investigated the presence of X recessive mutations possibly causing male sterility. We found that the gene at one limit of the sex locus, annotated as *INAPERTURATE* (*INP1)* [21], showed a 8-bp deletion in exon2 in all X haplotypes, resulting in a premature stop codon in its DOG domain (involved in DNA binding) and a truncated protein (Fig. 3b). We also found that four genes were absent from the X haplotype (Figs. 1a, 2, and 3a, Additional file 1: Fig. S2). They included a gene previously annotated [6] as a short homolog of Ethylene-overproducer-like-1 (*ETOL1)*. Three other genes were also absent from the X haplotype, namely a gene encoding a short peptide of unknown function (Unk), *NAP1* and *UAP56A*.

### Finding candidate female-sterility genes

Second, we searched for dominant mutations causing female sterility in the Y haplotype and for possible mechanisms of reversion to hermaphroditism. The Y-specific genes described above (*INP1*, *ETOL1*, *UNK*, and *UAP56*) are not expressed at the stage where ovule abortion occurs and are unlikely candidates for female sterility (Additional file 1: Fig. S7). Therefore, we searched for differential expression of X and Y alleles in XY gene pairs. Three genes showed higher total expression in males than in females in the sex locus: *VviPLATZ*, *VviFSEX*, and *APRT3* (Fig. 4a). *VviPLATZ* and *APRT3* showed a similar expression pattern in males, with a twofold induction during bud development. In females, *APRT3* expression dropped down from early to late flower development. *VviPLATZ* expression was very low at all stages in females. Remarkably, only the Y alleles of *VviPLATZ* and *APRT3* (*VviPLATZy* and *APRT3y*) showed a significant induction in male buds (Fig. 4b, Additional file 1: Fig. S8). *VviFSEX* showed a decreasing versus stable expression in female and males, respectively. Expression of the X and Y alleles were similar in males.

If these genes are involved in female sterility in males, they should have a modified expression in hermaphrodites. It is the case of all three genes (Fig. 4a). Even though *VviPLATZy* has a similar expression pattern in both hermaphrodites and males, it is expressed at a lower level all over the floral development in the former than in the latter (Fig. 4b). Both *VviFSEX* alleles show similar expression in hermaphrodites but contrary to males, their expression tend to decline during floral development similarly to females. In sharp contrast with males, *APRT3y* was not expressed in hermaphrodites, making it a particular interesting candidate female sterility gene. *APRT3* is the ortholog of a gene encoding an enzyme of cytokinin elimination. We found that *APRT3x* is expressed both in female and male buds, especially at early stages, and is a ubiquitously expressed gene in grapevine (Additional file 1: Fig. S9). We found that all analyzed cultivars possessed at least one *APRT3x* allele (recombinant *APRT3x/APRT3x* genotypes were observed but no *APRT3y/APRT3y*, Fig. 1b).

## Discussion

### The wild grapevine genome and sex locus

Compared to the available *V. vinifera* reference genome (486 Mb, 2061 scaffolds, 14,634 contigs with a N50 of 102 kb, 26,346 genes and more than 41,000 proteins, see https://www.ncbi.nlm.nih.gov/genome/401?genome_assembly_id=214125), the *V. sylvestris* diploid reference genome that we generated here has similar size (469 Mb), fewer contigs (591), and a larger N50 (1.7 Mb). Both genomes have a chromosome-level assembly and are of high quality (Additional file 2).

In agreement with previous work in *V. vinifera*, we found that the sex locus is on chromosome 2 of *V. sylvestris* too. Our study shows that it is 111 kb long, smaller than the lower bound estimates from previous work. Picq et al. found that *YABBI*, a gene close to the sex locus, was not fully genetically linked to it [6]. This was confirmed here. The non-recombining region extends from *TPP* to *APRT3*; the genes outside of this region are recombining and are not part of the sex locus. They may be part of the sex determination gene network but not as master genes. Note that we use sex locus, sex-determining region, Y-differentiated region, and non-recombining region of the sex chromosomes as synonyms here as in previous work in grapevine. The region that we delineated might however not include sex-determining genes only but also other genes unrelated to sex.

### Molecular evolution of the grapevine sex locus

We found that the divergence between the X and the Y haplotypes is low but using a molecular clock for plants and assuming a generation time from 5 to 52.5 years, we found that the sex locus may be between 10.7 and 112.5 MY old. These numbers may appear very high given the low dS we found for the sex locus. However, our results are consistent with the *Vitis* genus being slow-evolving [28, 29]. The radiation the *Vitis* genus (including subgenera *Vitis* and *Muscadinia*) from a dioecious ancestor took place 28 to 54 MY ago [30, 31]. Previous work suggested that the *V. sylvestris* sex locus might be shared with other *Vitis* species [4, 6]. A recent study has identified the sex locus in several *Vitis* species and found it to be conserved in both subgenera [32]. If we assume that the sex locus is 28–54 MY old, as suggested by these observations, using our molecular clock, we obtain an average generation time of 13.1–25.2 years for *V. sylvestris*, which is not unrealistic. All these pieces of evidence converge to the idea that the grapevine sex locus is relatively old, at least as old as the *Vitis* genus itself and perhaps older.

Previous works have emphasized the importance of inversions (usually on the Y) in suppressing recombination between sex chromosomes (e.g., [22, 24, 33]). Here we did not find evidence for inversions between the X, Y, and Yh haplotypes of the grapevine sex locus. Recombination must have been suppressed through another mechanism. Transposable elements are known to be abundant in non-recombining regions, which was also observed here in the Y haplotype of the *V. sylvestris* sex locus, but TE accumulation is usually seen as a consequence of the absence of recombination, not a cause [34, 35]. Unfortunately, recombination modifiers are poorly known in plants [36]. It will be interesting to explore this in future research.

### A genetic model for the sex determination of wild and cultivated grapevines

**Fig. 5:**
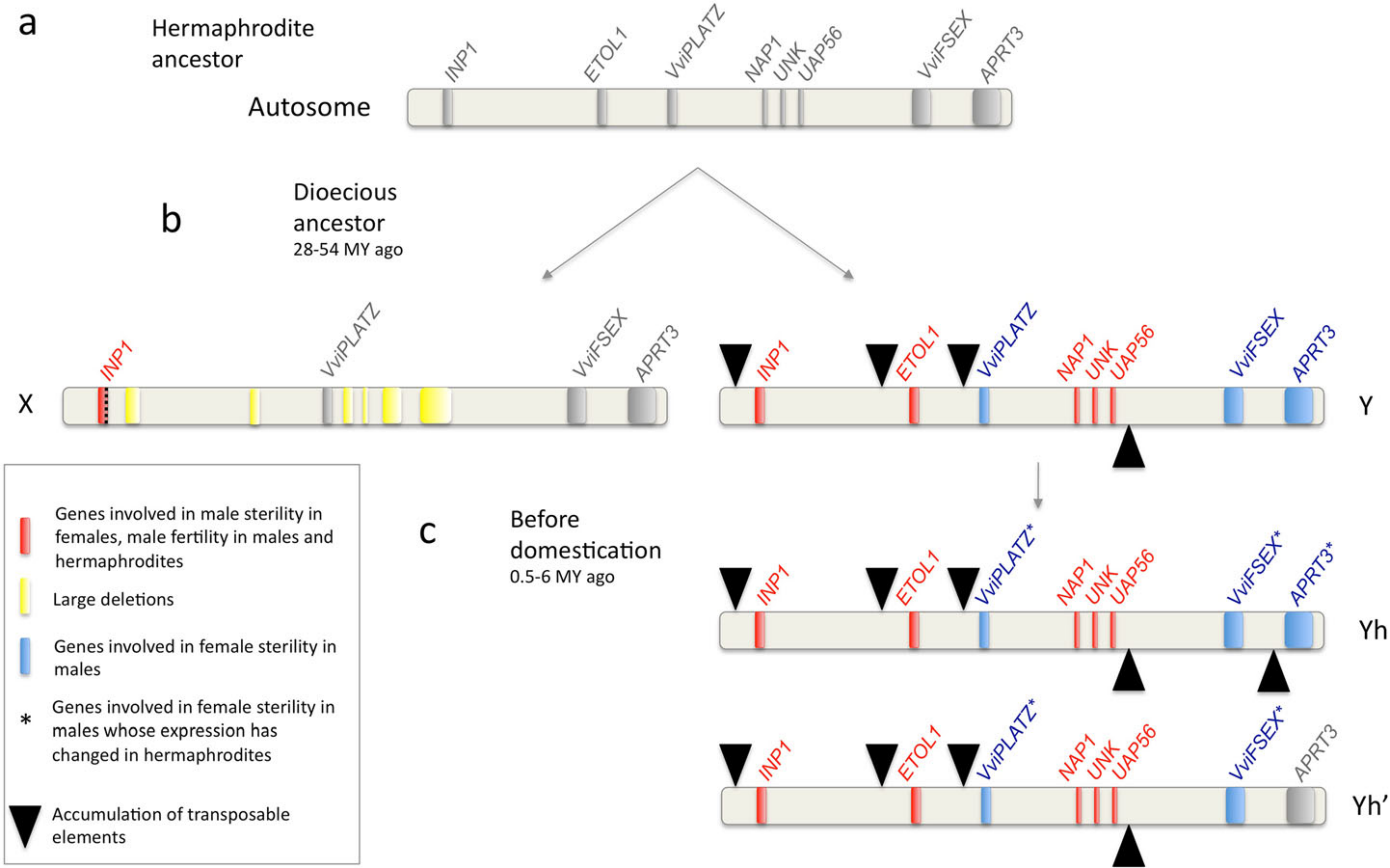
Evolutionary genomic scenario of the formation of the X and Y haplotypes and reversal to hermaphroditism. **a** Reconstructed gene content and order in the hermaphroditic ancestor (before the evolution of dioecy). **b** Formation of the X and Y haplotype in the dioecious ancestor of all *Vitis* species. **c** Formation of the Yh haplotypes through a modification of the expression of the genes involved in female sterility (standard Yh) and/or by a rare recombination event at the *APTR3* gene (Yh′)

In the classical genetic models, two genes with a dominant suppressing ovule allele (*So*) and a recessive suppressing pollen one (*sp*), respectively, explain sex determination in grapevine [3]. When searching for *sp*, we identified five candidates: *INP1*, *ETOL1*, a short peptide of unknown function, *NAP1*, and *UAP56A*. *INP1* loss-of-function mutant in *Arabidopsis thaliana* lacks pollen apertures [37], similarly to the pollen of female *V. sylvestris* [2, 3]. We found that *INP1* expression was highly specific of mature flower buds of *V. vinifera*, consistent with a role in late pollen development [38]. Inaperturate sterile pollen evolved independently at least six times in eudicots in association with dioecy [39]. It remains to be shown however that the absence of apertures in grapevine female pollen is sufficient to cause sterility in grapevine. Some of the *A. thaliana INP1* knock-out mutants may be fertile [38], while those mutants in maize are infertile [40]. *ETOL1* shows homology to *NPG1*, which is essential to pollen germination in *A. thaliana* [41], and the ethylene pathway has been shown to be determinant in floral morph determination in *Cucumis melo* [42]. *NAP1* encodes a nucleosome assembly protein in *A. thaliana*. Two homologs of *UAP56A*, a DEAD-box ATP-dependent RNA helicase, have been shown to regulate programmed cell death during tapetum development in *Oryza sativa* [43], and disrupting them led to male sterility. None of these three genes were significantly expressed in the early developmental stages of the RNA-seq dataset that were produced to study ovule development (Additional file 1: Fig. S7), likely because male sterility occurs later than female sterility in *V. sylvestris* flowers [27]. *INP1* and the four X-deleted genes were all present in at least one haplotype in cultivars. These results suggest that several recessive deletion mutations affecting genes involved in tapetum and pollen development may cause the male sterility syndrome (Fig. 5). We were not able to identify genes that could explain why the non-functional anthers observed in females are reflexed.

When searching for *So*, we identified three candidates: *VviPLATZ*, *VviFSEX*, and *APRT3*, a gene involved in the cytokinin pathway*.* Exogenous application of cytokinin is sufficient to restore female fertility in male *V. sylvestris* [44]. Previous in situ hybridization work suggested that APRT3 was induced during male bud development and absent in female buds [9]. We suggest that *APRT3x* performs essential functions in both *V. sylvestris* and *V. vinifera*. In contrast, *APRT3y* is specifically activated in males, suggesting that this allele form may trigger a decrease in cytokinin concentration in the carpel and cause its abortion (Figs. 4c, d and 5). Downregulation of *VviPLATZy*, *APRT3y*, and perhaps *VviFSEX* may be sufficient to cause reversal to hermaphroditism (Fig. 4, Additional file 1: Text S1). In addition (but not in a mutually exclusive way), transition to hermaphroditism may be caused by recombination upstream of the *APRT3* promoter leading to the *APRT3x/APRT3x* genotype that is observed in several cultivars (Figs. 1b and 5).

We found a great functional coherence in *V. sylvestris* sex locus with tapetum and pollen genes involved in male-sterility and cytokinin pathway genes involved in female-sterility. Pollen and phytohormone pathway genes have previously been involved in sex determination [45, 46].

### Evolution of sex determination in plants: insights from grapevine

The dominant theoretical model for the evolution of dioecy and sex chromosomes in plants relies on two sex-determining genes, carrying, one, a dominant female-sterility mutation and, the other, a recessive male-sterility mutation [47]. However, other models are possible [45, 46, 48]. In some species such as wild strawberries, *Asparagus*, date palms and kiwifruit, two candidate sex-determining genes have been found in agreement with the two-gene model [49–52], while in other species such as persimmons and possibly cucurbits, only one gene seems to be determining both male and female fertility [53–56].

Here we found several candidate sex-determining genes, which seems to rule out the single-gene model in grapevine. However, more than two candidates were identified, which suggest that the evolution of sex determination in grapevine might not have followed exactly the simple two-gene model in which a recessive male-sterile mutation and a dominant female-sterile mutation successively spread in the population evolving towards dioecy. We identified several possible male-sterile mutations, and female-sterility is possibly mediated by several genes of the sex locus. Despite its small size, the sex locus is rather enriched in pollen and ovule genes, it is possible that sex determination initially evolved with two of these genes, and was reinforced later by secondary genes. However, the X and Y haplotypes’ gene contents are conserved among *Vitis* and *Muscadinia* subgenera [4, 6, 32]. This suggests that all the sex-determining genes evolved in the dioecious ancestor of the whole *Vitis* genus (and possibly before).

Duplication and neofunctionalization have played important roles in the evolution of sex determination in all dioecious plants in which sex-determining genes have been identified [49–53]. In grapevine, we found that male sterility may have evolved through full or partial deletions as in other systems [49, 51, 52]. However, the evolution of the female sterility may have implied the expression divergence of the Y allele compared to the X allele of several genes (the X alleles conserving the ancestral function), a mechanism of neofunctionalization of the Y-allele, not documented before in dioecious plants.

How the expression of *ViviPLATZy*, *VviFSEXy*, and *APRT3y* was modified remains to be elucidated. Their cis-regulatory regions may have gained some new transcription factor binding sites [32]. Transposable elements may also have played a role. We indeed found accumulation of TEs close to *ViviPLATZy*, which may have influenced its expression patterns (Figs. 2 and 5). Remarkably, the most important accumulation of TEs in the Yh compared to its Y progenitor was upstream of the *APRT3* gene, whose expression is strongly modified in hermaphrodite. TE insertions are known to lead to silencing of nearby genes, as in the case of the genes controlling sexual forms in monoecious cucurbits [57].

### Changes in the sex locus and grapevine domestication

In hermaphrodites, *ViviPLATZ*, *VviFSEX*, and *APRT3* expression is modified. The modification of *APRT3* expression was the most drastic (Figs. 4 and 5), and it is possible that it is sufficient to restore female sterility. It is interesting to note that the main structural variation between Y and Yh also lies at *APRT3*. In one Yh haplotype, the *APRT3* upstream region includes many TE insertions (as discussed above). Another Yh haplotype (Yh′) found on Chardonnay [21] was formed by the recombination between a Y and a X at *APRT3*, which basically formed a Y haplotype with a *APRT3x* (Fig. 5). The Yh haplotypes are thus only slightly modified compared to the Y one. This may suggest that the switch to hermaphroditism during domestication was easy. This is in agreement with recent studies suggesting that dioecy is rare in flowering plants because it has a high reversion rate to hermaphroditism [58]. Hermaphrodites often occur naturally in dioecious plants [59]. Hermaphroditic *V. sylvestris* have already been observed in the wild [60] [3]. The Yh haplotypes may have pre-existed domestication as a standing natural variation. Our estimates of the age of the Yh haplotype (based on dS values) indeed support this idea. It is possible that the domestication process started with hermaphroditic individuals. It is also possible that the Yh haplotype was introgressed in the *V. vinifera* (possibly several times independently) from *V. sylvestris* or other *Vitis* species late during the domestication process, a scenario that is consistent with the complex phylogenetic relationship between the X, Y, and Yh haplotypes from different cultivars and wild populations of *V. sylvestris* [6].

## Conclusions

Our work provides a comprehensive characterization of the sex locus in *V. sylvestris*, a model for the sex determination in *V. sylvestris* and the switch to hermaphroditism in *V. vinifera* and a new reference genome, which is a valuable genomic resource to study diversity and domestication in grapevine. Future genetic and functional studies will be needed to dissect the role of the candidate genes that we found in different aspects of the male sterility syndrome, female sterility, and reversal to female fertility in hermaphrodites. It will be also interesting to understand better the evolution of the grapevine sex locus: how recombination was suppressed between the X and Y haplotypes. The study of the sex locus in many cultivated and wild grapevine populations may help understand more precisely the switch to hermaphroditism.

## Methods

### Sequencing data

#### Whole-genome sequencing (PacBio—long reads) of Sylvestris C1-2

The accession of Sylvestris C1-2 with plant code 8500.Col.C1-2, origin of the geographic location Sainte-Croix-en-Plaine, Haut-Rhin (68), France, was used for extraction of high-molecular-weight genomic DNA. The extraction was performed by the CNRGV (Centre National de Ressources Génomiques Végétales) – INRAE Toulouse. They started with 1G of frozen material (− 80 °C), crushed it with liquid nitrogen, and used the Genomic-tip 100/G kit (Qiagen) for the extraction. The SMRT library preparation and sequencing on PacBio RSII platform (P6-C4 chemistry) was done by the IGM Genomics Center at the University of California, San Diego, following the standard PacBio protocols. A total of 129X coverage was obtained in order to perform de novo genome assembly.

#### Whole-genome sequencing (Illumina—short reads) of Sylvestris C1-2

DNA libraries with two different library size from leaves of Sylvestris C1-2 were prepared in order to perform 2 × 151 paired-end short reads sequencing (Illumina). One library had a library size of 740 bp (860 bp with adapters), and the other library had a library size of 392 bp (512 bp with adapters). The two libraries were sequenced on the same flowcell lane. The libraries preparation and Illumina sequencing was performed by the EPGV (Etude du Polymorphisme des Génomes Végétaux) – INRAE. A total of 338,109,086 reads was obtained (~ 100X coverage) for all samples together, used to polish the Sylvestris C1-2 PacBio assembly.

#### RNA-sequencing of Sylvestris C1-2

RNA-sequencing was performed on six samples of the Sylvestris C1-2 accession. Three biological replicates of whole green berries and three biological replicates of whole mid-ripening berries were sequenced. The RNA-seq library preparation was performed with the TruSeq Stranded mRNA Library Prep Kit (Illumina) and was sequenced in paired-end (2 × 100 bp) on HiSeq4000 platform (Illumina technology). Library preparation and sequencing was performed by the GenomEast platform—Strasbourg. A total of 1,113,531,260 reads were obtained for all samples together, used to assemble a Sylvestris transcriptome and perform genes annotation.

#### Whole-genome re-sequencing of a cross between C1-5 (female *Vitis sylvestris*) and Martigny_2 (male *Vitis sylvestris*)

These two accessions were crossed in INRAE Colmar ampelographic collection. The accession C1-5 female *Vitis sylvestris* (plant accession number at INRAE Colmar 8500.Col.C1-5) originated from the geographic location Sainte-Croix-en-Plaine, Haut-Rhin (68), France, and the male accession Martigny_2 (plant accession number at INRAE Colmar 8500.Col.1) was an accession originating from the geographic location Martigny, Swiss. The progeny was grown at INRAE Colmar greenhouses. The DNA samples of both parents, of five male descendants (44613.Col.5026T, 44613.Col.5028T, 44613.Col.5029T, 44613.Col.5033T, and 44613.Col.5053T) and five female descendants (44613.Col.5035T, 44613.Col.5040T, 44613.Col.5046T, 44613.Col.5050T, and 44613.Col.5057 T), were sequenced. The quantity and quality of the DNA extracted from leaves (DNeasy plant mini kit, Qiagen) of 12 individuals from the cross (2 parents and 10 offspring) was checked prior to sequencing. Nanodrop analysis indicated that concentrations were always above 110 ng μl^−1^, and that more than 5 μg DNA was available for all samples. Quality (fragment size) was checked using capillary electrophoresis (Fragment Analyzer) and was satisfactory. Based on quality check, we adapted sonication (using Covaris E220) in order to get mostly fragments of 250 bp. Twelve Illumina libraries were constructed and were pooled for two lanes of sequencing on an Illumina Hiseq 4000 machine in 2 × 100 bp paired-end mode. This yielded between 85 and 149 million reads per individual (Additional file 1: Table S2), which roughly corresponded to 17X to 30X coverage of re-seq data per individual.

### Genome assembly, cleaning, polishing, anchoring, and ordering

Shortly, the Sylvestris C1-2 de novo genome assembly was performed with Falcon-integrate (Falcon + Falcon-unzip), in order to obtain a diploid assembly (the two haplotypes). With Falcon, PacBio reads were self-corrected, assembly was performed, haplotypes were generated, assembly was polished with Arrow, and finally we obtained a phased diploid assembly of Sylvestris C1-2.

#### Genome assembly and phasing

The FALCON-integrate 1.8.4 tool used is available on github: https://github.com/PacificBiosciences/FALCON-integrate/tree/1.8.4. The FALCON parameters used for the *Vitis sylvestris* genome assembly are taken from the genome assembly of *V. vinifera* cv Cabernet Sauvignon paper [10] (pa_HPCdaligner_option = -v -dal128 -e0.75 -M60 -l2500 -k18 -h1250 -s100 -w8; ovlp_HPCdaligner_option = -v -dal128 -M60 -e.96 -l1500 -s100 -k24 -h1250; pa_DBsplit_option = -a -x500 -s200; ovlp_DBsplit_option = -s200; falcon_sense_option = --output_multi --min_idt 0.70 --min_cov 4 --max_n_read 400 --n_core 8; falcon_sense_skip_contained = False; overlap_filtering_setting = --max_diff 120 --max_cov 120 --min_cov 4 --bestn 10 --n_core 8). The phasing and haplotypes creation was performed with Falcon-unzip, with default parameters. The assembly was polished with FALCON and PacBio reads using Arrow (available in Falcon-integrate).

#### Assembly polishing and finishing

After the FALCON run, we performed additional polishing with Illumina reads. First, Illumina reads were aligned with bwa [61] mem and -M option on FALCON’s genome assembly. Then, alignments were filtered with samtools to keep only primary alignments and concordant pairs. Finally, alignments were filtered with bamtools to keep alignments with an edit distance ≤ 5. These filtered aligned reads are used to polish the assembly with the pacbio-util (version 0.2) from pacbio-utilities tool (https://github.com/douglasgscofield/PacBio-utilities). Then, the same alignment was performed on the genome polished with pacbio-utils, and this genome was polished with Illumina reads and PILON (v1.22 - https://github.com/broadinstitute/pilon). A few haplotigs may have remained in the primary contigs file. A tool is available, purge_haplotigs, to find these false primary contigs in order to move them to the haplotigs file. We used this tool to correct this and to finish our genome assembly (v1.0.4 - commit 6414f68 - https://bitbucket.org/mroachawri/purge_haplotigs/src/master/).

#### Quality control of the assembly

Assembly statistics such as number of contigs, N50 and L50, were calculated with a home-made script. Genome assembly completeness was assessed with BUSCO [62] with the genome mode, the embryophyta_odb9 lineage, and the *Arabidopsis* species options (version 2.0 - https://gitlab.com/ezlab/busco). Nucmer tool (from MUMmer tool: https://sourceforge.net/projects/mummer/ - nucmer (version 3.1) parameters: -maxmatch -l 100 -c 500) and the grapevine reference genome [5] (PN40024, version 12X.2 - https://urgi.versailles.inra.fr/Species/Vitis/Data-Sequences/Genome-sequences) were used to align the Sylvestris assembly to the PN40024 reference assembly in order to see completeness of this Sylvestris assembly. Nucmer and grapevine reference genome were also used to anchor and order Sylvestris contigs into chromosomes (with additional parameters –r –q), as it was done in the Chardonnay genome assembly paper [63].

### BAC generation and sequencing

#### Generation of BAC libraries of a *Vitis sylvestris* and Cabernet Sauvignon to obtain the sex locus Y, Yh, and X sequences

We first chose a *V. sylvestris* male from a wild population spontaneously growing on a hill forest near Montpellier (France), on the Northern slope of the Pic Saint Loup mountain. Its male phenotype was confirmed over 5 years of observation of the flowers, both on the forest plant and on 5 of its clones planted in the INRAE Vassal Grape Collection in Marseillan, France (introduction name: Lambrusque PSL10; introduction number: 8500Mtp107). High molecular weight DNA was isolated from 40 g of PSL10 young leaves following the cell nuclei extraction method described in [64] with slight modifications [65]. The long DNA fragments were partially digested with EcoRI restriction enzyme, and fragments from 100 to 250 kb were selected. Sized and eluted DNA was then ligated into a pAGIBAC-EcoRI cloning vector and cloned into DH10B T1R *Escherichia coli* strain (Invitrogen). The resulting BAC clones were plated on a solid selective medium and organized in barcoded microplates using a robotic workstation QPix2 XT (Molecular Devices). The BAC library was named Vsy-B-Lamb. It consists of 27,648 clones of 113 kb size in average, representing a 6.4x genome equivalent coverage. The library was replicated for security reason, and the two copies were stored in separate freezers at − 80 °C.

We also used an existing BAC library of Cabernet Sauvignon (Cabernet Sauvignon clone 412, ENTAV-INRA®), available at CNRGV Toulouse, France, named VVCS-CabernetSauv. Both libraries are today available at CNRGV: https://cnrgv.toulouse.inrae.fr/fr/Banques/Vigne.

#### BAC library screening

The bacterial clones were deposited on a macroarray nylon membrane (22 × 22 cm), following a 6 × 6 grid pattern. Three copies of this gridded macroarray were created. To select BAC clones carrying the DNA fragments from the sex locus, we used specifically designed radio-labeled probes. These probes were defined using the sequences published in [6]: VSVV006, VSVV007, VSVV009, VSVV010, VSVV011 (GeneID GSVIVT01001275001, GSVIVT01001277001, GSVIVT01001286001, GSVIVT00007310001, GSVIVT00007312001 respectively). On the Vsy-B-Lamb BAC library, the hybridization of 3 separate pools of probes allowed to spot around 200 putatively positive BAC clones in total; the 30 clones showing the most intense spots were individually tested via real-time PCR using the same sequences used for probe design and 9 clones were validated. The same clustering of the 9 positive clones into two groups of alleles was obtained using two approaches: the first assignation based on their melting temperature curves similarities, and the second based on Sanger sequencing of internal and BAC-end sequence polymorphisms. These two groups correspond to the two alternate alleles expected in a “XY-like” sex region. Internal and BAC-ends sequences were also used to map the BACs on each other, for each allele, with the objective to sequence the minimal number of clones with an optimized overlapping to cover the whole region (minimum tilling paths composed of 3 and 4 clones respectively). The same procedure was used on the VVCS-CabernetSauv library to spot the sex-locus BACs and assign them respectively to the X and Yh allele, Yh designating modified Y haplotypes found in hermaphrodites.

#### BAC sequencing and assembly of X, Y, and Yh haplotypes

The sequences of the 7 sex-region BACs of *V. sylvestris* and the 6 BACs of Cabernet Sauvignon were obtained via a PacBio RSII sequencer (P6C4 chemistry). Sequencing was done in a pool of 20 individually tagged BAC clones. We used 2 μg of each individual BAC clone DNA to prepare the PacBio SMRT® 10 kb library. GeT-PlaGe Genomic Platform (INRAE-Toulouse, France) handled the sample loading on the RSII device and the data retrieval. We performed the detection and removal of residual *E. coli* sequences on raw reads. After a second cleaning step consisting in detecting and removing the vector sequences, individually tagged BAC sequences were assembled with the HGAP workflow (https://github.com/PacificBiosciences/Bioinformatics-Training/wiki/HGAP).

For each of the groups of BACs identified as above (X, Y, and Yh), the BAC sequences were joined using their overlapping, so to form long “haplotigs”. For *Vitis sylvestris*, one haplotig was then assigned to the “Y haplotype” and the other to the “X haplotype” using the sex-discriminating polymorphisms described by [6], namely 10 SNPs for VSVV006, 7 SNPs for VSVV007, and 6 SNPs for VSVV009. These SNPs were found to be 100% associated with sex, in a worldwide collection of 22 males, 23 hermaphrodites, and 91 females [6], and we confirmed that the two haplotigs had either all male SNPs or all female SNPs. We used a similar procedure for calling X and Yh alleles from Cabernet Sauvignon BACs sequencing. Our Cabernet Sauvignon haplotig assemblies were also confirmed by comparison with those publicly available at that time, with a sequence identity of 99.97% over 260 kb, the few differences being all located in mononucleotide repeats ([20], see http://169.237.73.197/CabSauv/).

### Genome and BAC annotation

#### Genome annotation

RNA Illumina paired-end reads obtained from grape berries of Sylvestris C1-2 (see above) were used to assemble a transcriptome in order to annotate the genome. Prinseq-lite [66], Ribopicker [67], and STAR [68] were used to clean the data, and the assembly was made using Trinity [69]. We assembled 398,189 putative transcripts (422,652,106 bp), with a N50 of 2217 bp, which roughly corresponds to the average size of Pinot noir transcripts (2056 bp). TransDecoder [69] detected 187,734 CDS with an average size of 832 bp (of which 60,985 CDS are larger than 832 bp). A BUSCO analysis detected 1237 genes, of which 1166 (94%) are complete. EuGene-EP [16] (Eukaryote Pipeline - v1.4 - http://eugene.toulouse.inra.fr/) was used to perform genes annotation on the Sylvestris assembly. The protein databases used for the genes annotation were the TAIR10, swissprot, and uniprot plants databases. The transcriptomes used for the genes annotation were our Sylvestris transcriptome, 812 manual annotated genes, and transcript sequences of *V. vinifera* from NCBI - 2017-11-08. The configuration file with all the parameters used is available as supplementary data (egnep. Vvi.for_Sylvestris.cfg). Primary contigs and haplotigs were annotated separately, with the same parameters. Finally, we obtained two gene annotations, one for primary contigs and one for haplotigs, with different files: gene annotation in a gff3 file, gene/mrna/cds/ncrna/protein sequences in separate fasta files, and some statistics per gene.

#### BAC annotation

The BAC sequences’ annotation (*V. sylvestris* Y haplotype P2, *V. sylvestris* Y haplotype P1, *V. sylvestris* X haplotype, *V. vinifera* Cabernet Sauvignon Yh and X haplotype sequences and the annotation with EuGene [16] (Eukaryote Pipeline - v1.4 - http://eugene.toulouse.inra.fr/)) was started with the same parameters and reference files than for the whole-genome annotation. The protein databases used for the genes annotation were the TAIR10, swissprot, and uniprot plants databases. The transcriptomes used for the genes annotation were our Sylvestris transcriptome, 812 manual annotated genes, and transcript sequences of *V. vinifera* from NCBI - 2017-11-08. The configuration file with all the parameters used is available as supplementary data (egnep. Vvi.for_Sylvestris.cfg). Then, the annotations specific to BACs were extracted and used as final BACs’ annotation. We did not run Eugene on the 5 BAC sequences alone because EuGene learns from data, and we did not want to introduce a bias due to the low number of BAC sequences. Annotation of both full and partial fragments of transposable elements was carried out on the same BAC sequences with RepeatMasker 4.1 [17]

### Development of SEX-DETector++

We developed a new version of the SEX-DETector software that implements a probabilistic method to study SNPs segregation in a family, taking into account genotyping errors. SEX-DETector++ is coded in C++ and uses additional algorithmic optimization to reduce the running time and memory usage by about two orders of magnitude compared to the original code. SEX-DETector++ thus allows convenient usage on large, genome-wide genotyping datasets for which the original code would require prohibitively large running times and memory. The underlying model is the same as in the original code, but new functionalities were added to deal with genomic data as an input of the method (in the vcf format). The code is publicly available at https://gitlab.in2p3.fr/sex-det-family/sex-detector-plusplus, where technical and installation details can also be found.

### Characterization of the sex-linked region in the *V. sylvestris* genome

#### SNP-tolerant mapping and SNP discovery from whole-genome re-sequencing data

Divergence between X and Y alleles can prevent SNP discovery. To avoid this issue, we carried out an iterative SNP-tolerant mapping similarly to the procedure described in [14]. Raw reads of twelve individuals of the same family (2 parents and 5 descendants of each sex) were first mapped against the *V. sylvestris* genome with gsnap [70] v2018-07-04 (-m 0.1) in standard mode. At each iteration step, SNP calling was performed: variants were called with samtools mpileup v1.3.1 [71] followed with Varscan v2.4.3 [72] mpileup2snp (Min coverage 8, Min reads2 2, Min var. freq 0.2, Min avg. qual 15, *P* value thresh 0.01). Additional filters were applied: minimum frequency of variants reads between 0.25 and 0.75 for heterozygote genotypes in individual samples, maximum coverage of twice mode of the Gaussian distribution for each sample, only bi-allelic SNPs, minor frequency variant higher than 0.05, and maximum rate of missing data 0.2. A SNP database was built with gsnap utilities to perform a SNP-tolerant mapping at the next iteration. We carried out two iterations of SNP-tolerant mapping until the rate of discovery of new XY SNPs (see below) became low. Finally, a 4th step of SNP-tolerant mapping with more stringent parameters (-m 0.05, primary alignments only) was performed in order to reduce the rate of false-positive SNPs.

#### Detection and validation of sex-linked single nucleotide polymorphisms

In order to detect SNPs linked to the sex-determining region, we run SEX-DETector++. This yielded 4113 sex-linked SNPs (with a posterior probability high than 0.6), 90.5% of them on chromosome 2. As the sex-determining regions is assumed to be small [4, 6] and the low number of offspring in our cross gives us access to few recombination events, the sex-linked region in our analysis may appear larger than the actual sex-determining region (SNP close to the sex locus tend to be linked to sex in a particular cross). To determine the boundaries of the sex-determining region, independent public data were analyzed in order to identify shared XY SNPs between different populations. WG-reseq and RNA-seq data from wild and cultivated grapevines were mapped against the *V. sylvestris* genome with gsnap v2018-07-04; SNPs were called with varscan v2.4.3. We searched for candidate XY-SNPs that were heterozygote in all male samples and homozygote in all female samples of the validation dataset. Validated XY-SNPs spanned 111 kb on chromosome 2 (from 4,810,929 to 4,921,949 bp), which indicated the boundaries of the sex-determining region. In order to complement SEX-DETector++ analysis, we also performed an empirical one. We searched for SNPs that overlapped the sex-determining region and were homozygote in female individuals of the cross and heterozygote in male individuals, allowing two missing alleles. This empirical analysis retrieved all 1406 XY SNPs identified by SEX-DETector++ plus 459 additional ones (+ 32.6%). This final dataset of 1865 XY SNPs was used for downstream analyses.

#### Determination of the synonymous divergence between X and Y alleles in the sex-linked region

The dataset of XY SNPs was used to build X and Y allelic pseudosequences in coding regions, using a custom python script to substitute reference positions by X or Y SNPs respectively in the genome in respect with strand, and to extract and concatenate coding DNA sequences for each gene of the sex locus. The yn00 program of the PAML suite was used to estimate synonymous divergence (dS) with standard error estimation [73]. The same procedure was used to calculate the dS between the Y *V. sylvestris* haplotype and the Yh haplotype of Cabernet Sauvignon.

#### Structural characterization of the sex-linked region in the *V. sylvestris* genome

Structural gene annotations in the sex-linked regions were extracted from Eugene (see annotation section). Predicted proteins were mapped against the NCBI nr database with BLASTP, and functional annotations were manually analyzed. The best hit in *Arabidopsis thaliana* was used to name the genes. Transposable elements were detected with Red [74] and RepeatMasker 4.1 [17].

### Integrative search for sex-determining genes in the sex locus

#### Comparison of gene and TE content in X, Y, and Yh haplotypes

In addition to the female whole genome of *V. sylvestris*, we used the assembled BAC sequences of X and Y haplotypes of *V. sylvestris* and Yh and X haplotypes of Cabernet-Sauvignon. BLASTN (ncbi-blast v2.2.3) were carried out between pairs of haplotypes (X-Y, X-Yh, Y-Yh, from whole-genome and from BACs). Whole-haplotype comparisons were visualized with CIRCOS [75] to identify presence/absence patterns. Genic sequences were extracted in all haplotypes using Eugene annotations and BLASTN, and aligned with mafft v7 online service [76] to identify structural differences and mutations. The sequences of the putative sex-determining candidates were blasted against assemblies of other cultivated grapevine genomes (Chardonnay, Cabernet-Sauvignon and Zinfandel) to assess their presence or absence.

#### Total and allele-specific expression of sex-linked genes

We retrieved raw reads from 23 public libraries of female, male, and hermaphrodite bud flowers’ samples from *V. sylvestris* at four developmental stages [27]. Reads were mapped on the *V. sylvestris* genome with gsnap v2018-07-04 (-m 0.1), with a mode tolerant to XY SNPs. Read counts were obtained with htseq-count v0.10.0 [77] and normalized by computing the RPKM (reads per count per millions of reads mapped). To specifically measure the expression of X and Y alleles, we performed SNP calling (gsnap –m 0.05, varscan max missing data 0.4 and minor allele frequency 0.05) and extracted the positions corresponding to XY SNPs located in CDS from the vcf file. For each gene and library, we read the number of reads mapping on reference and variant alleles (corresponding to X and Y alleles respectively) with bedtools intersect [78]. Reads numbers were summed by gene and normalized by the total number of reads in the variant file for the library and the length of transcripts.

#### Organ-specific expression of sex-linked genes in *V. vinifera*

We retrieved and mapped transcriptomic data of *V. sylvestris* and *V. vinifera* in several organs and conditions against the genome of *V. sylvestris* (berries; developing seeds; leaves under normal, drought, and pathogenic conditions; stem; early and mature flower buds; Additional file 1: Table S12). We measured the gene expression levels in the different conditions, and computed an index of organ-specificity (Tau) on normalized expression levels (log (read count per kilobase per millions of mapped reads)). The Tau specificity index ranges between 0 and 1 and typically display two modes near 0 and 1 indicating ubiquitous and organ-specific genes respectively [79].

### Statistical analyses and data visualization

Unless stated otherwise, statistical analyses were carried out in R v3.4.4 (2018-03-15) [80]. Data visualization was performed with R or with CIRCOS v0.69-6 [75]. The structural variation among the X, Y, and Yh haplotypes was represented using SimpleSynteny 4.1 (Veltri et al. 2016; https://www.dveltri.com/simplesynteny/). Adeneget was used to visualize SNP data in R [81].

## Supplementary information

**Additional file 1: Figures S1 to S9, Tables S1 to S12, Text S1.**

**Additional file 2.** Assembly statistics.

**Additional file 3.** List of X/Y SNPs.

**Additional file 4 **Transposable element positions on the X haplotype of the *V. sylvestris* genome.

**Additional file 5.** Transposable element content of the different BAC-derived haplotypes.

**Additional file 6.** Pairwise dS values of X vs Y and Y vs Yh haplotypes.

**Additional file 7.** Review History.

## Data Availability

All raw reads, genome assembly, and gene annotation are available in ENA under the bioproject PRJEB37020 [82]. The source code of SEX-DETector++ is available at the url https://gitlab.in2p3.fr/sex-det-family/sex-detector-plusplus [83] available under the GNU General Public License v3.0 and archived at Zenodo [84].
