# Supplementary material for: The wild grape genome sequence provides insights into the transition from dioecy to hermaphroditism during grape domestication
Source: Genome Biol. 2020 Sep 7;21:223. doi: 10.1186/s13059-020-02131-y (PMC7487632; doi:10.1186/s13059-020-02131-y)
Supplement: Supplementary file 1 — Additional file 1: Figures S1 to S9, Tables S1 to S12, Text S1. [file 13059_2020_2131_MOESM1_ESM.pdf]

Supplementary Information

Table of contents

Supplementary Figures..2

Supplementary Tables..14

Supplementary Text..28

Supplementary Figures

Supplementary Figure 1: BUSCO results on *V. sylvestris* pseudomolecules. Out of 1440 genes in the BUSCO dataset, 95% are complete.

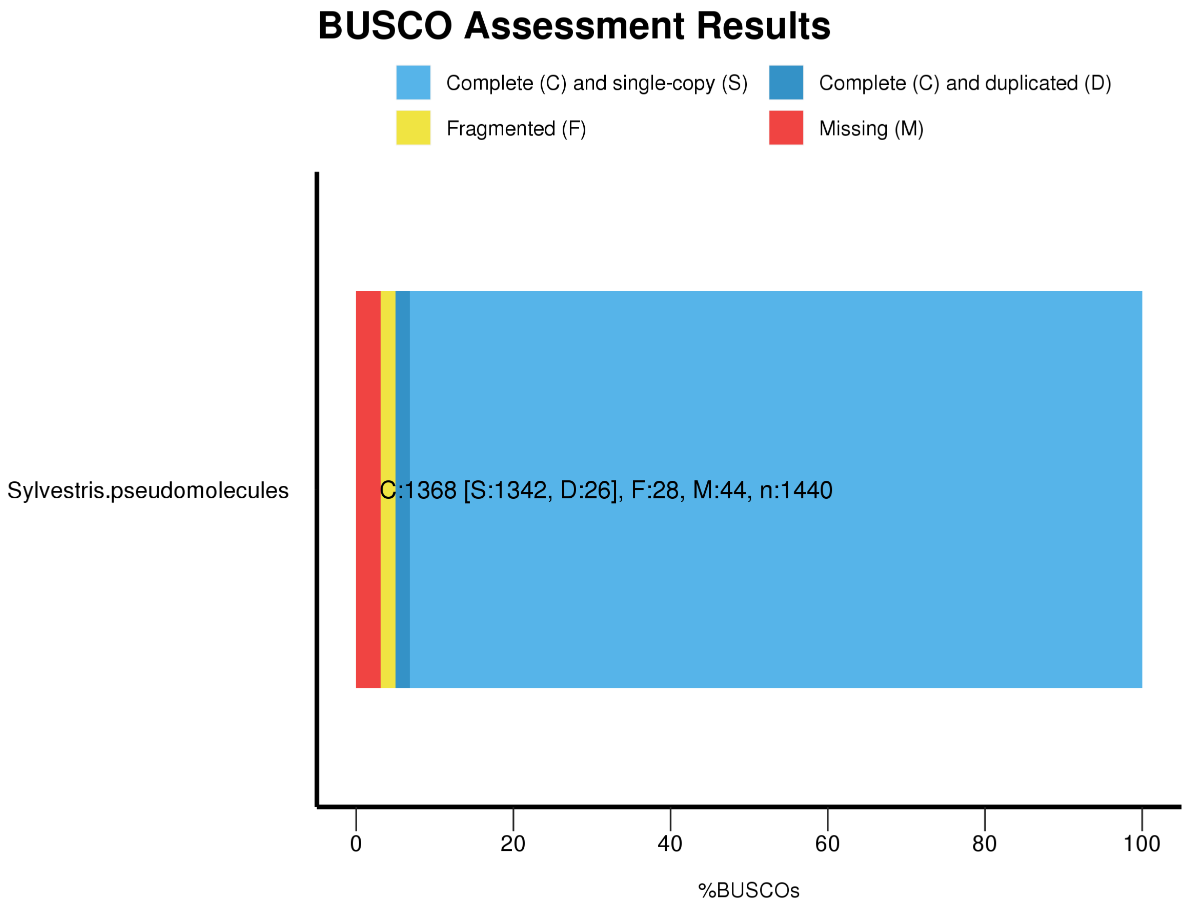

Supplementary Figure 2: Structural comparison of X and Y haplotypes of the sex locus of *V. sylvestris*. Outer to inner track: circular representation of pseudomolecules; limits of genes (obtained from Eugene and verified with blastn) in grey and repeats in green; synteny relationships (blastn hits with an e-value lower than 0.01. YP1 and YP2 are two BAC contigs covering the Y locus, with a gap of an estimated size of 13kb, in which presence of the *PLATZy* allele has been confirmed by PCR (see Supplementary Text). Coordinates are indicated in Mbp.

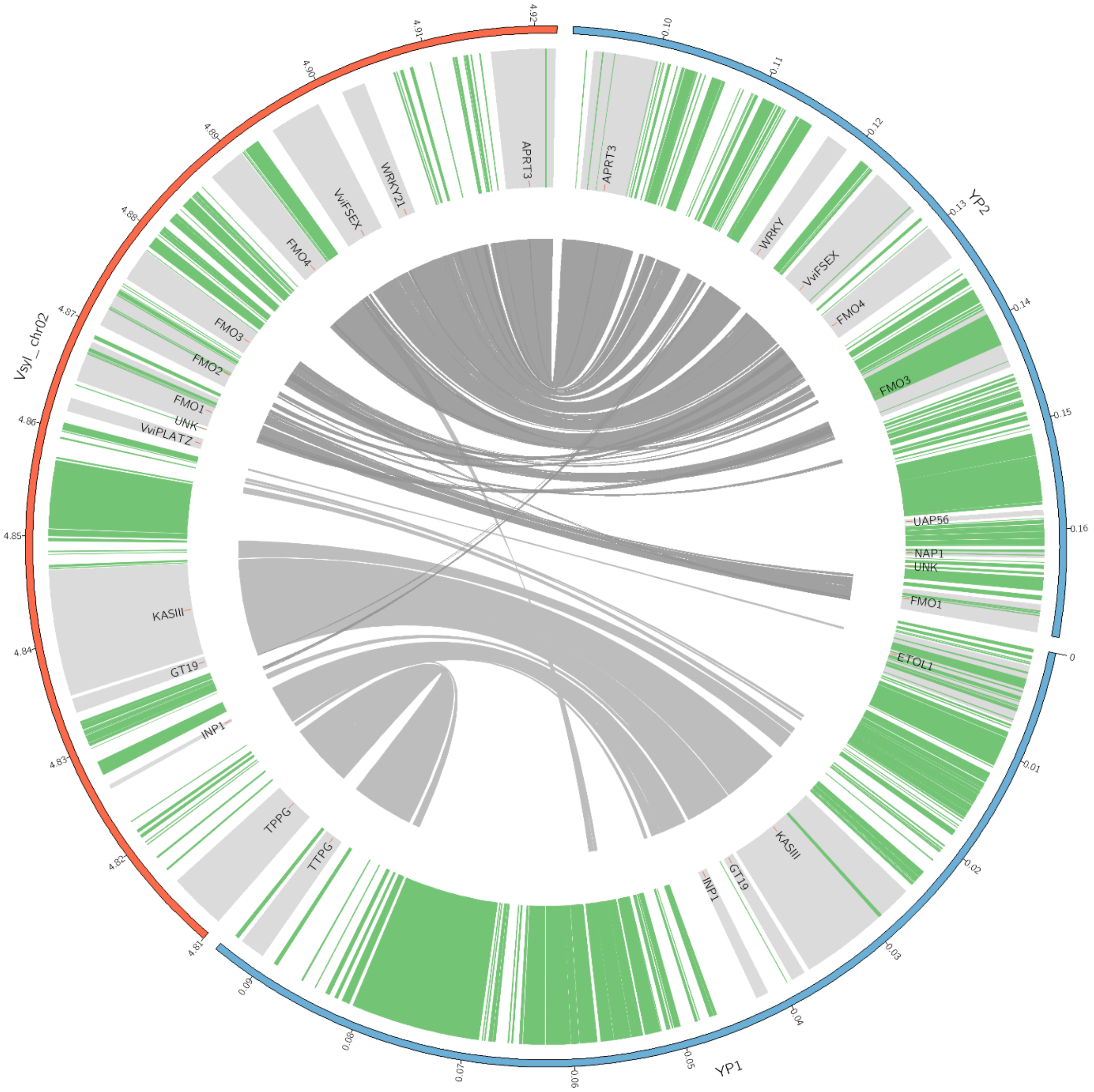

Supplementary Figure 3: Density of XY-SNPs and location of X-hemizygote regions in a cross in *V. sylvestris*. Track, form outter to inner track: genomic coordinates on chromosome 2 (from 4.810 to 4.922 Mb), location and name of genes (blue = direct strand, red = reverse strand), Depth of mapping coverage in a male individual (RCDN9), from 0 to 50, Depth of mapping coverage in a female individual (RCDN16), number of XY SNPs (from 0 to 64). All values were computed by 1-kb overlapping windows. The number of XY SNP was not normalized in order to reflect the proportion of informative sites in each window. Yellow highlights represent the approximate limites of X-hemizygote regions inferred from a two-fold reduction of mapping coverage in males.

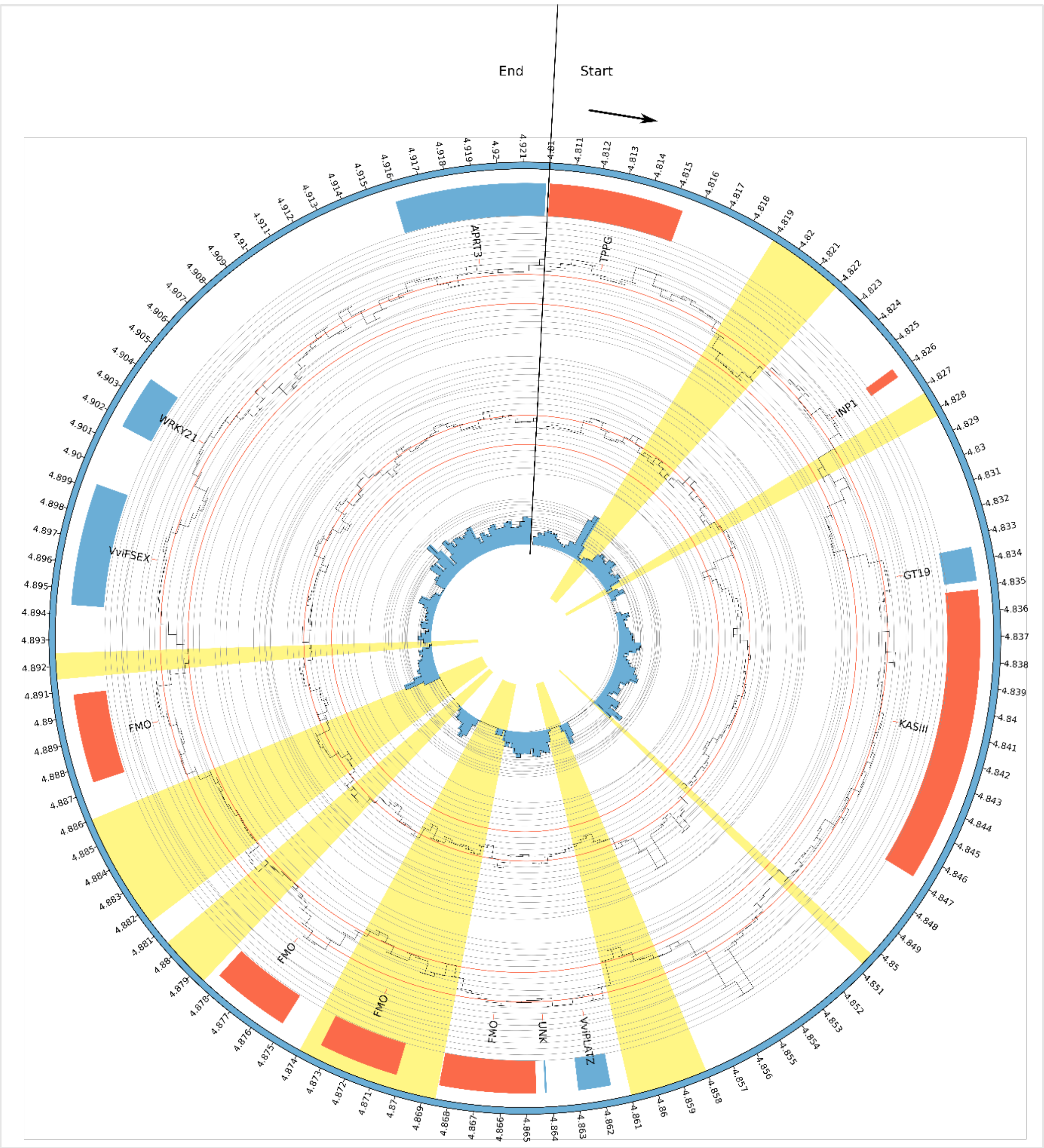

Supplementary Figure 4: Structural comparison of a Y haplotype of *V. sylvestris* and a Yh haplotype of *V. vinifera* cv. Cabernet Sauvignon, Yh referring to the modified Y haplotype found in hermaphrodites. Outer to inner track: circular representation of pseudomolecules; limits of genes (obtained from Eugene and verified with blastn) in grey and repeats in green; synteny relationships (blastn hits with e-values lower than 0.001). YP1 and YP2 are two BAC contigs covering the Y locus, with a gap of an estimated size of 13kb, in which presence of the *PLATZy* allele has been confirmed by PCR (see Supplementary Text). The purple ideogram represents the Yh haplotype. A large insertion between *WRKY* and *APRT3* is present in the Yh haplotype of Cabernet Sauvignon, but is not shared by all Yh haplotypes (*e.g.* only of the two Yh haplotypes of Chardonnay, not shown). Coordinates are indicated in Mbp.

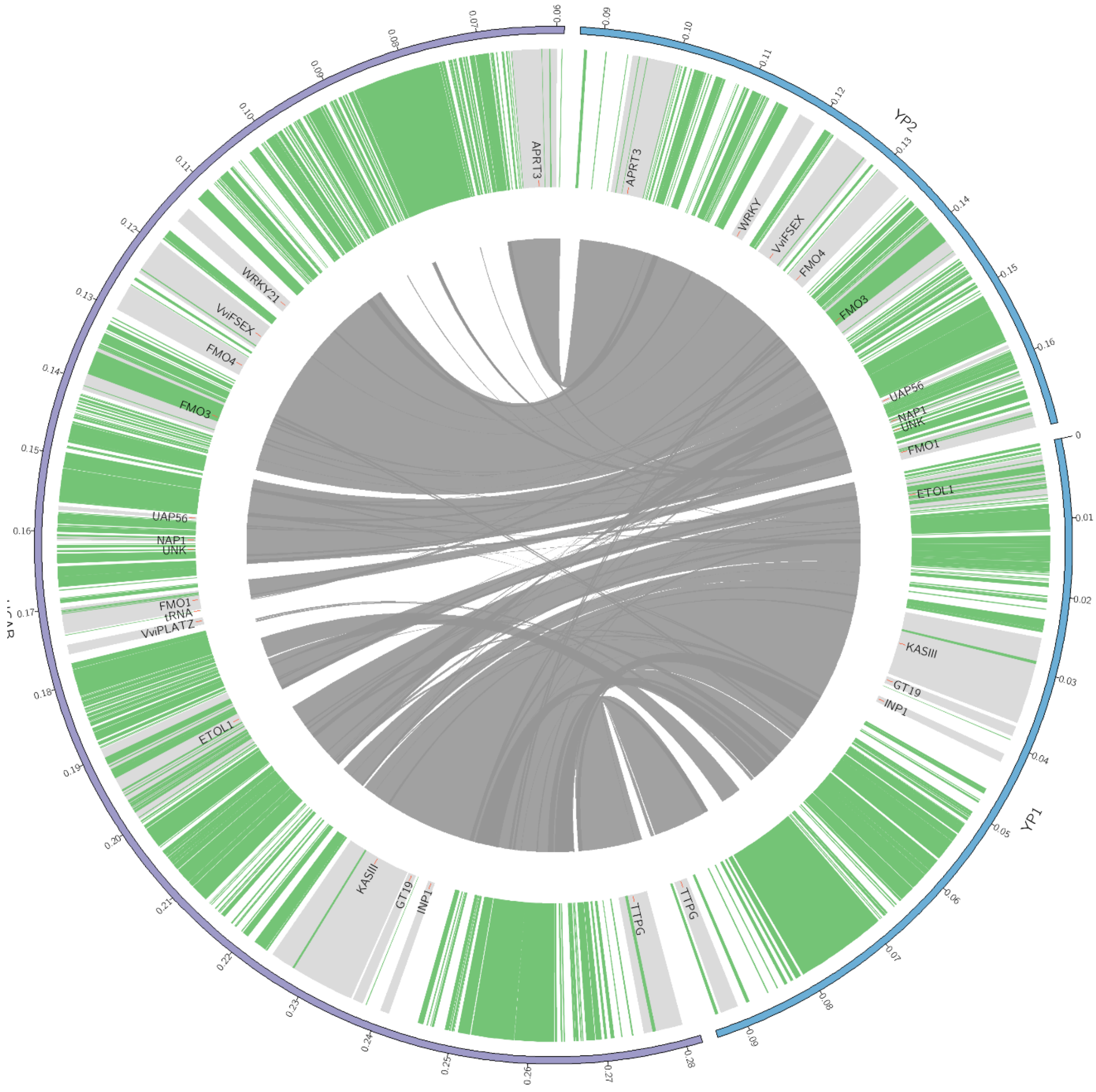

Supplementary Figure 5: Structural comparison of X haplotype of *V. sylvestris* and a Yh haplotype of *V. vinifera* cv. Cabernet Sauvignon. Outer to inner track: circular representation of pseudomolecules; Outer to inner track: circular representation of pseudomolecules; limits of genes (obtained from Eugene and verified with blastn) in grey and repeats in green; synteny relationships; synteny relationships (blastn hits with e-values lower than 0.001). Coordinates are indicated in Mbp.

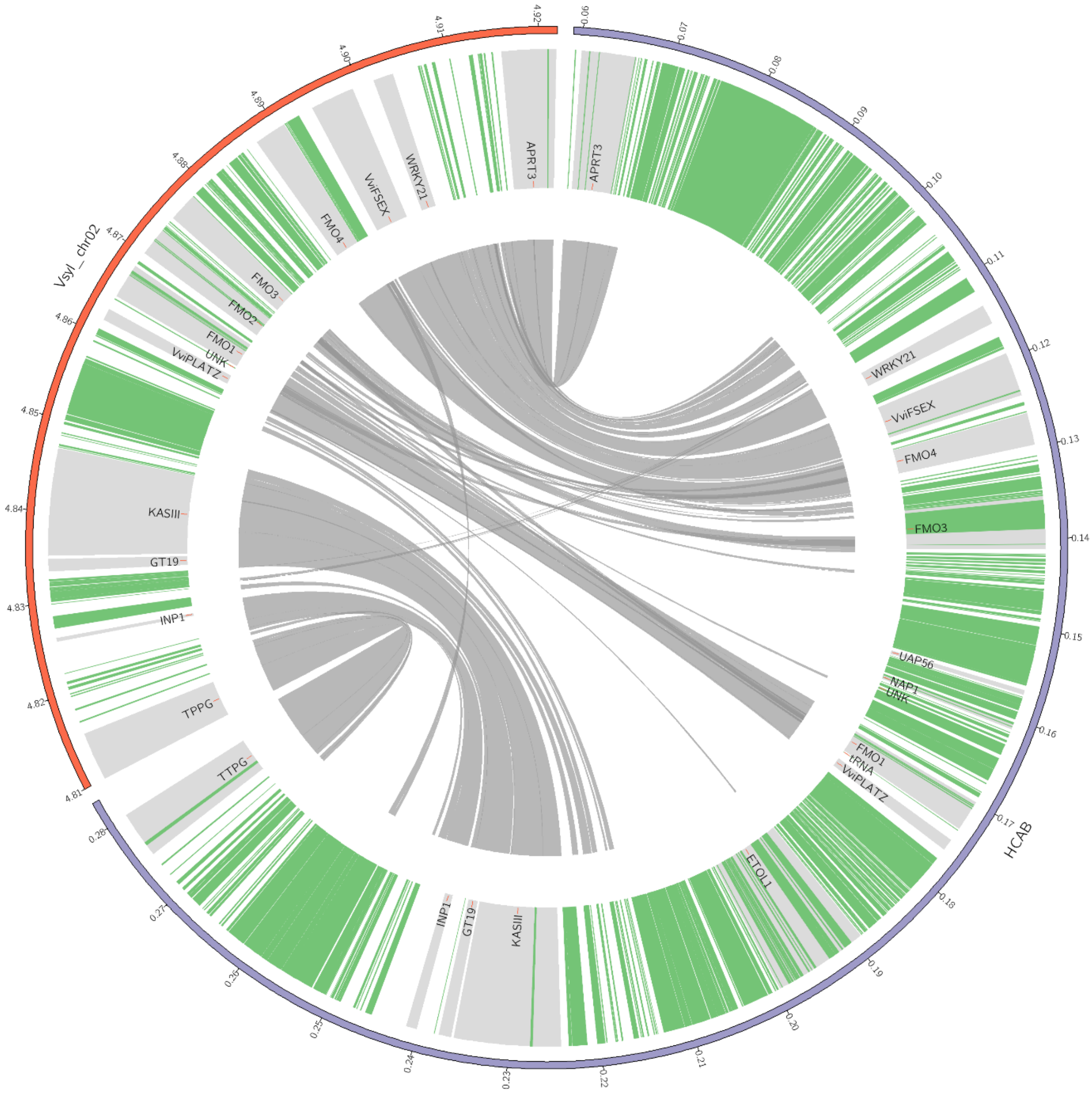

Supplementary Figure 6: Dotplots of BAC sequences, for each haplotype, demonstrate colinearity and absence of large size inversions on the region covering the 111kb sex linked region (dashed line, approx.) and extending outside the locus. X and Y axes represent base pairs. A. X and Y haplotypes of the *V. sylvestris* PSL10. B. *Vitis sylvestris* Y haplotype and Cabernet sauvignon X haplotype. C. Cabernet sauvignon Yh haplotype and *Vitis sylvestris* Y haplotype.

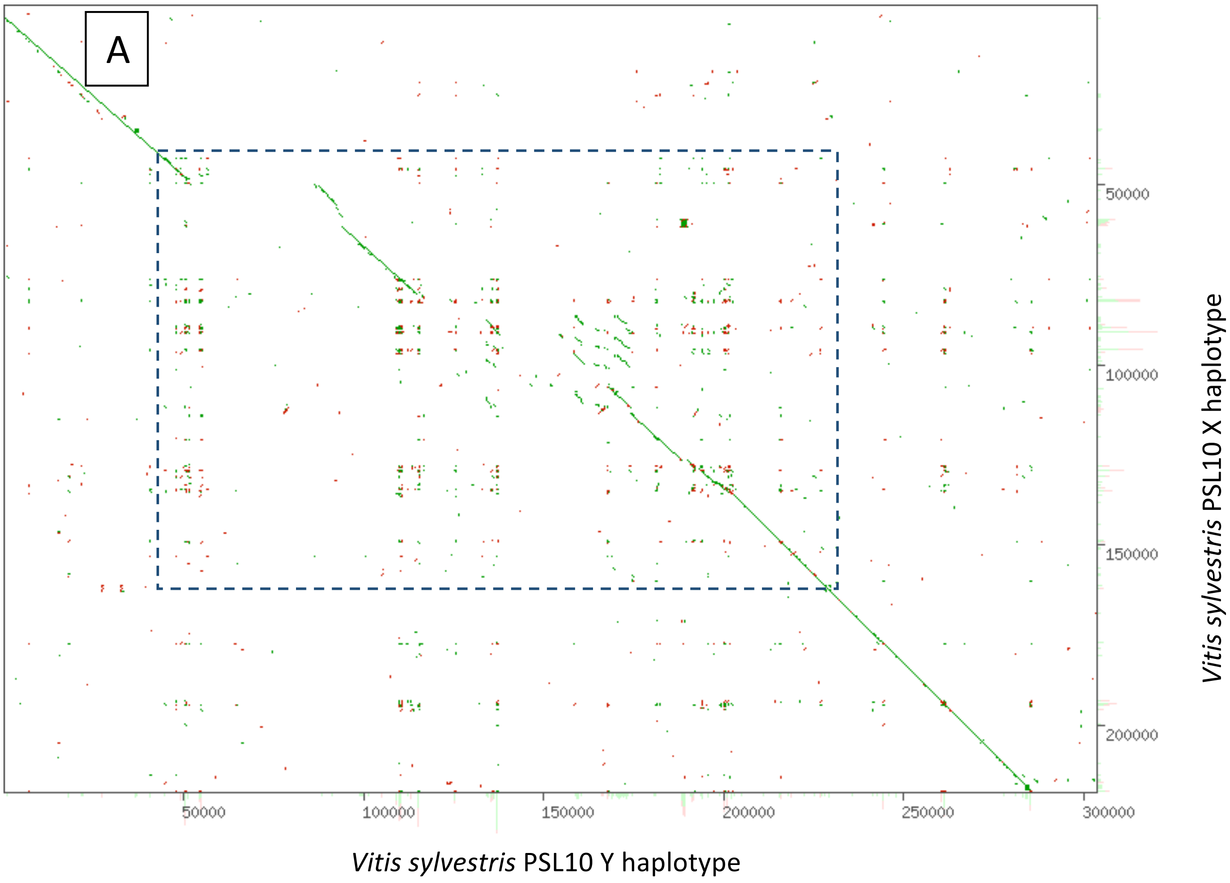

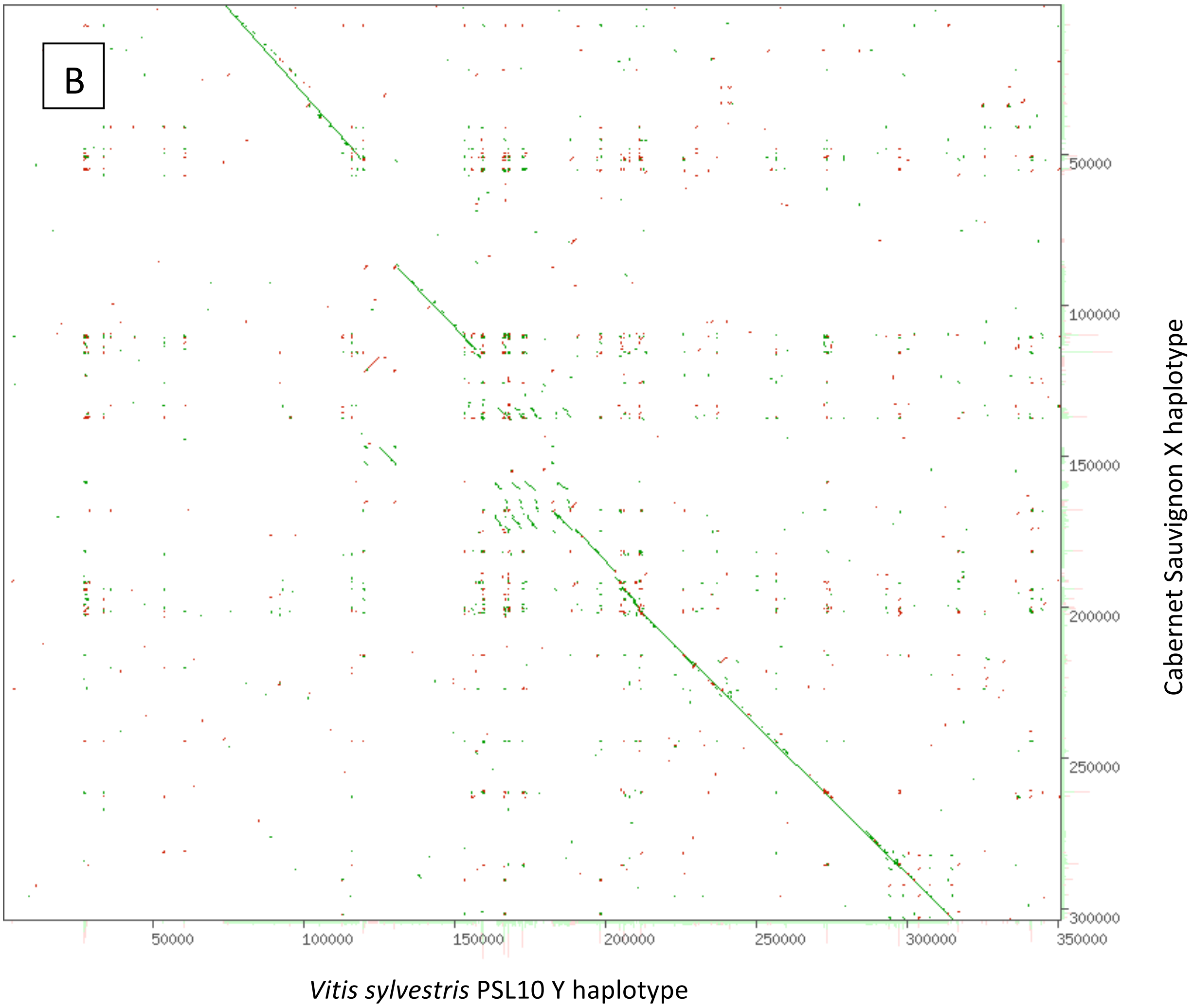
B
50000
100000
150000
200000
250000
300000
350000
Vitis sylvestris PSL10 Y haplotype
Cabernet Sauvignon X haplotype

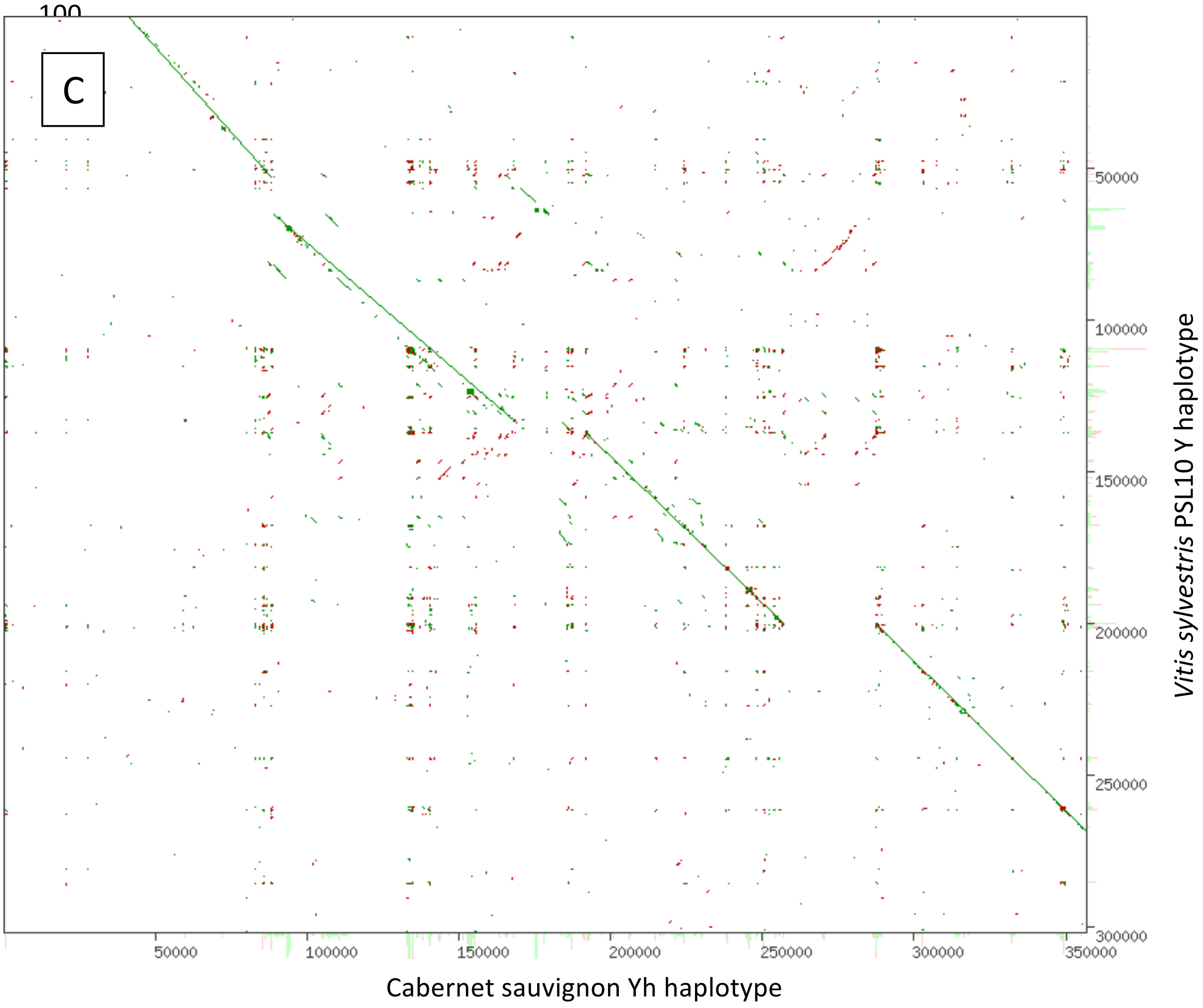
C
50000
100000
150000
200000
250000
300000
350000
Cabernet sauvignon Yh haplotype
50000
100000
150000
200000
250000
300000
Vitis sylvestris PSL10 Y haplotype

Supplementary Figure 7: Total normalized gene expression in males, females and hermaphrodites of *V. sylvestris* for genes in the sex locus during flower bud development. Letters B to H refer to successive developmental stages. Expression in females, males and hermaphrodites are represented by orange, blue and purple colors, respectively.

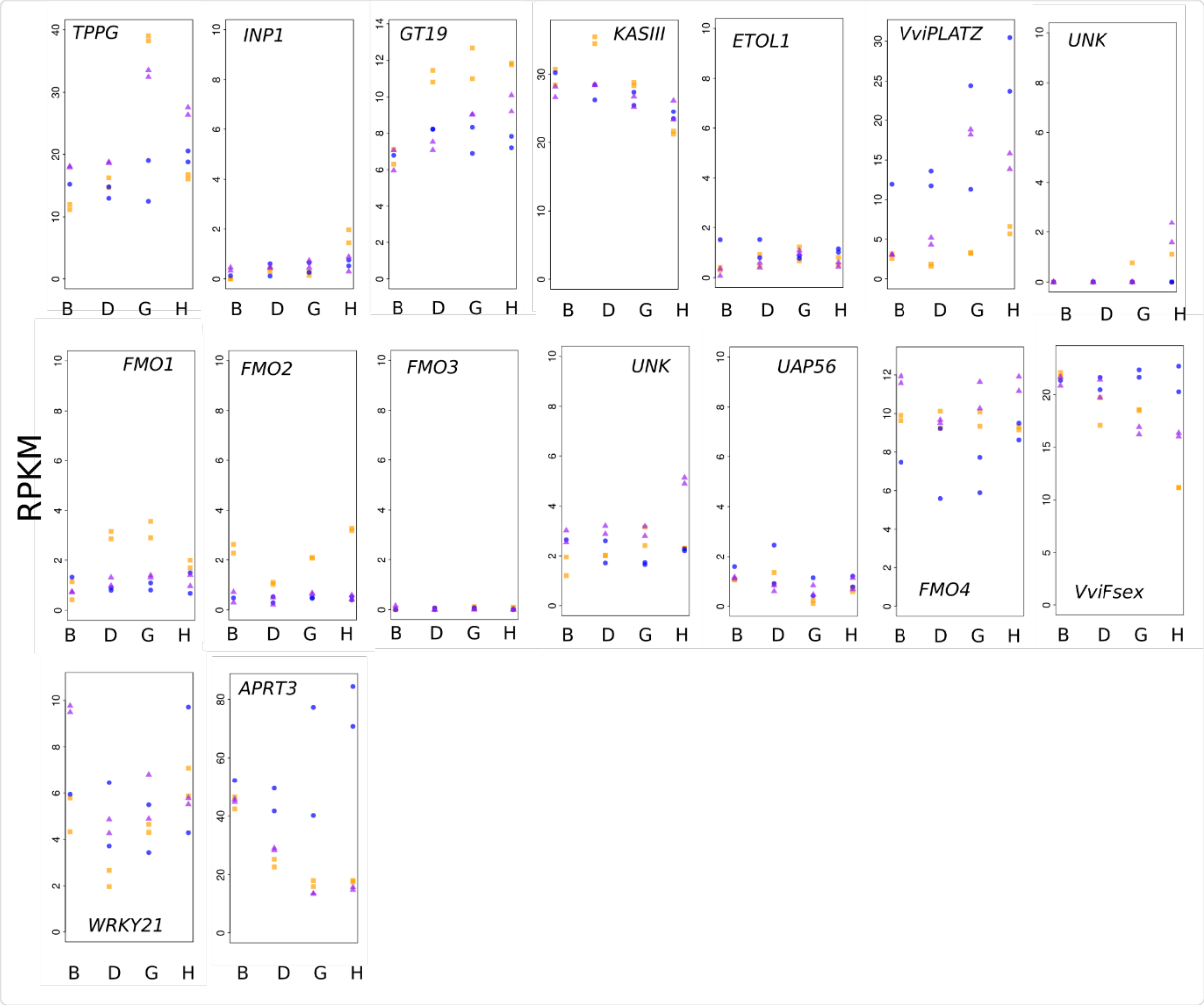

Supplementary Figure 8: Allele-specific expression of X and Y alleles of XY genes pairs of *Vitis sylvestris* during the development of flower buds. The X and Y allelic expression from B to H-stages flower buds is shown for females, males and hermaphrodites. Orange triangles, blue dots and grey diamonds represent X, Y and total allelic expression respectively. Allele-specific expression was computed only for genes with sufficient mapping coverage on at least 5 XY SNPs. The y scale is different for each gene but shared for a given gene between females, males and hermaphrodites. Two replicates are represented for each condition, except for the male B stage for which there was no replicate.

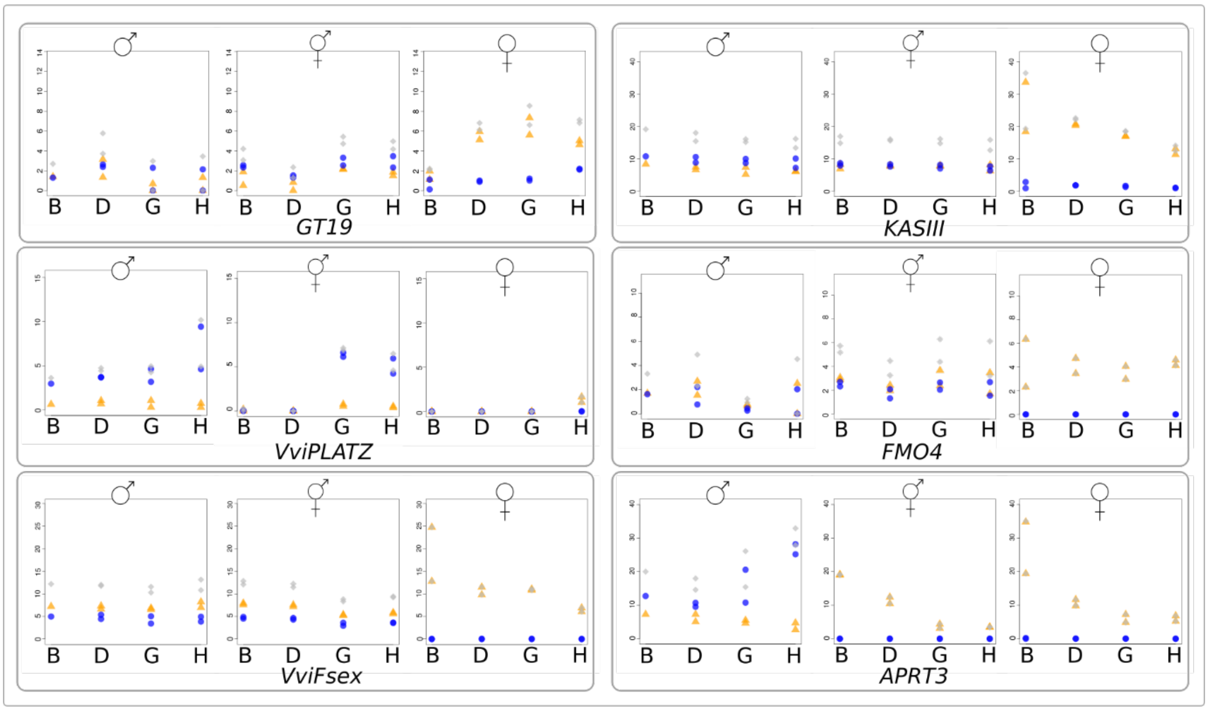

Supplementary Figure 9: Distribution of organ-specificity gene expression. The value of the specificity index Tau for four genes of the sex locus in indicated by vertical lines. Figure prepared using data shown in Supplementary Table 12.

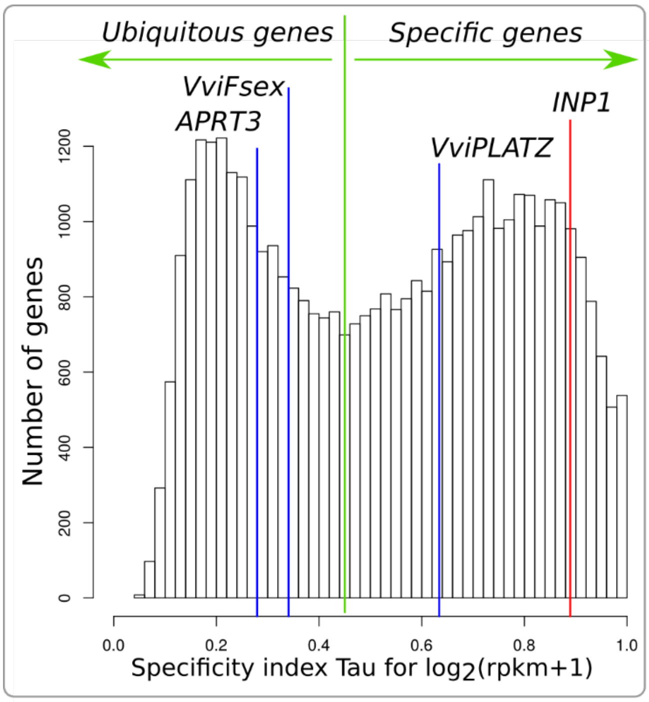

Supplementary Tables

Supplementary Table 1: Assembly and anchoring statistics of the *V. sylvestris* genome.

	Primary contigs	Anchored primary contigs	Haplotigs
Number of contigs	591	476	3,781
Mean length (b)	792,724	947,992	83,879
Median length (b)	421,056	584,494.5	36,553
Max length (b)	6,865,695	6,865,695	2,541,772
L50	82	77	483
N50 (b)	1,711,677	1,773,898	173,606
Assembly size (b)	468,500,071	451,244,463	317,149,633

Supplementary Table 2: Summary statistics of resequencing data of a cross between two *V. sylvestris* parents.

Sample ID	Genotype	Type	Sex	Concentraton ADN ng/µl	Number of Reads	Number of Pairs
RCDN8	C1-5	mother	Female	158	91,689,312	45,844,656
RCDN9	Martigny 2	father	Male	113	140,875,256	70,437,628
RCDN10	5026T	offspring	Male	150	149,542,818	74,771,409
RCDN11	5028T	offspring	Male	145	128,590,900	64,295,450
RCDN12	5029T	offspring	Male	145	85,976,170	42,988,085
RCDN13	5033T	offspring	Male	145	130,071,908	65,035,954
RCDN14	5053T	offspring	Male	155	123,960,284	61,980,142
RCDN15	5035T	offspring	female	110	109,564,824	54,782,412
RCDN16	5040T	offspring	female	110	132,609,212	66,304,606
RCDN17	5046T	offspring	female	130	111,932,706	55,966,353
RCDN18	5050T	offspring	female	110	106,023,554	53,011,777
RCDN19	5057T	offspring	female	120	125,195,484	62,597,742

Supplementary Table 3: Mode of the gaussian distribution of mapping coverage for each sample for the dataset of whole genome resequencing of a cross in *V. sylvestris*. The values are identical for each iteration of mapping and therefore indicated only once. The maximum coverage allowed corresponds to the maximum coverage that was allowed when filtering SNP, in order to remove repeated positions.

Sample ID	Mode of gaussian distribution	Maximum coverage allowed
RCDN8	16	32
RCDN9	25	50
RCDN10	26	52
RCDN11	22	44
RCDN12	14	28
RCDN13	22	44
RCDN14	21	42
RCDN15	19	38
RCDN16	23	46
RCDN17	19	38
RCDN18	18	36
RCDN19	22	44

Supplementary Table 4: Summary statistics of iterative SNP-tolerant mapping for the resequencing dataset of a cross in *V. sylvestris*.

Sample ID	Sex	Iteration 1			Iteration 2		
		Number of mapped reads	fraction of mapped reads	fraction of paired reads	Number of mapped reads	fraction of mapped reads	fraction of paired reads
RCDN8	F	89,951,765	0.981	0.969	90,089,021	0.983	0.971
RCDN9	M	138,338,630	0.982	0.971	138,544,634	0.983	0.973
RCDN10	M	146,671,291	0.981	0.968	146,904,980	0.982	0.971
RCDN11	M	125,913,524	0.979	0.966	126,122,927	0.981	0.969
RCDN12	M	84,214,280	0.98	0.967	84,352,679	0.981	0.969
RCDN13	M	127,437,731	0.98	0.967	127,645,009	0.981	0.969
RCDN14	M	121,369,732	0.979	0.966	121,578,081	0.981	0.968
RCDN15	F	107,360,998	0.98	0.967	107,538,009	0.982	0.97
RCDN16	F	130,211,584	0.982	0.971	130,408,313	0.983	0.973
RCDN17	F	109,719,948	0.98	0.968	109,900,170	0.982	0.97
RCDN18	F	104,161,079	0.982	0.971	104,315,801	0.984	0.973
RCDN19	F	122,729,069	0.98	0.967	122,927,332	0.982	0.97

Sample ID	Iteration 3			Iteration 4		
	Number of mapped reads	fraction of mapped reads	fraction of paired reads	Number of mapped reads	fraction of mapped reads	fraction of paired reads
RCDN8	90,095,720	0.983	0.972	90,029,816	0.982	0.97
RCDN9	138,552,746	0.984	0.973	138,452,568	0.983	0.972
RCDN10	146,916,364	0.982	0.971	146,801,740	0.982	0.969
RCDN11	126,131,254	0.981	0.969	126,036,403	0.98	0.968
RCDN12	84,358,053	0.981	0.969	84,294,600	0.98	0.968
RCDN13	127,653,125	0.981	0.969	127,558,702	0.981	0.968
RCDN14	121,585,814	0.981	0.968	121,490,591	0.98	0.967
RCDN15	107,545,495	0.982	0.97	107,458,696	0.981	0.968
RCDN16	130,416,024	0.983	0.973	130,319,644	0.983	0.972
RCDN17	109,907,858	0.982	0.97	109,819,091	0.981	0.969
RCDN18	104,322,423	0.984	0.973	104,244,590	0.983	0.972
RCDN19	122,935,210	0.982	0.97	122,840,655	0.981	0.969

Supplementary Table 5: Summary statistics of missingness per sample for each iteration of SNP-calling in the dataset of whole-genome resequencing of a cross in *V. sylvestris*. Statistics were obtained with vcftools version 0.1.15. N_MISS is the number of missing sites, F_MISS is the frequency of missing sites.

	Iteration 1		Iteration 2		Iteration 3		Iteration 4	
Sample ID	N_MISS	F_MISS	N_MISS	F_MISS	N_MISS	F_MISS	N_MISS	F_MISS
RCDN8	280,469	0.0685	257,255	0.0576	260,910	0.0557	259,983	0.0556
RCDN9	80,415	0.0196	78,141	0.0175	82,728	0.0177	82,577	0.0177
RCDN10	34,051	0.00831	45,378	0.0102	55,432	0.0118	57,368	0.0123
RCDN11	72,953	0.0178	79,836	0.0179	91,745	0.0196	90,777	0.0194
RCDN12	418,876	0.102	385,349	0.0863	397,437	0.0848	395,665	0.0847
RCDN13	59,795	0.0146	67,214	0.0151	77,745	0.0166	78,511	0.0168
RCDN14	81,071	0.0198	75,386	0.0169	80,678	0.0172	79,763	0.0171
RCDN15	112,434	0.0274	103,972	0.0233	110,945	0.0237	110,066	0.0236
RCDN16	51,669	0.0126	58,007	0.0130	66,040	0.0141	66,795	0.0143
RCDN17	112,887	0.0276	106,827	0.0239	114,224	0.0244	112,998	0.0242
RCDN18	138,484	0.0338	133,571	0.0299	143,660	0.0307	142,622	0.0305
RCDN19	54,255	0.0132	53,412	0.0120	58,227	0.0124	57,799	0.0124

Supplementary Table 6: Number of homozygous and heterozygous SNPs called at each iteration in the dataset of whole-genome resequencing of a cross in *V. sylvestris*. "% increase het step4/step1" indicates the percentage of increase in the number heterozygous SNPs detected between the first and fourth step of iterative mapping.

		Iteration 1		Iteration 2	
Sample ID	Category	Number of homozygous SNPs	Number of heterozygous SNPs	Number of homozygous SNPs	Number of heterozygous SNPs
RCDN8	M	380,285	1,674,962	428,741	1,915,267
RCDN9	F	763,168	1,868,596	813,700	2,079,174
RCDN10	S	419,471	2,336,345	449,943	2,581,917
RCDN11	S	447,082	2,129,876	481,153	2,359,784
RCDN12	S	376,336	1,784,861	429,508	2,032,012
RCDN13	S	411,888	2,172,007	443,658	2,407,092
RCDN14	S	446,637	2,141,363	486,468	2,390,231
RCDN15	D	411,587	2,004,887	451,048	2,242,332
RCDN16	D	441,713	2,240,436	475,968	2,482,939
RCDN17	D	388,490	2,099,888	426,892	2,352,568
RCDN18	D	394,402	2,152,081	434,417	2,406,320
RCDN19	D	397,321	2,055,753	429,151	2,293,760

	Iteration 3		Iteration 4			
Sample ID	Number of homozygous SNPs	Number of heterozygous SNPs	Number of homozygous SNPs	Number of heterozygous SNPs	% increase hom step4/step1	% increase het step4/step1
RCDN8	442,840	2,061,120	442,698	2,062,008	16.41	23.11
RCDN9	832,308	2,241,277	830,372	2,242,601	8.81	20.02
RCDN10	460,069	2,756,124	459,710	2,757,158	9.59	18.01
RCDN11	494,110	2,522,773	491,744	2,524,116	9.99	18.51
RCDN12	443,293	2,175,229	442,599	2,174,875	17.61	21.85
RCDN13	454,834	2,569,706	453,889	2,569,619	10.20	18.31
RCDN14	499,287	2,557,644	498,176	2,559,111	11.54	19.51
RCDN15	463,449	2,400,577	462,964	2,400,871	12.48	19.75
RCDN16	487,714	2,654,078	486,224	2,655,479	10.08	18.53

RCDN17	438,941	2,512,766	439,365	2,516,241	13.10	19.83
RCDN18	447,979	2,565,013	447,227	2,567,305	13.39	19.29
RCDN19	438,548	2,459,524	438,145	2,460,816	10.27	19.70

Supplementary Table 7: Whole-genome resequencing dataset of the ncbi short read archive (SRA) that were mapped to the *Vitis sylvestris* genome. All samples were part of study SRP108271. Sample SRS2238702 had a lower mapping coverage than other samples, resulting in a high rate of missing data.

SRA run ID	Sample Accession	Domestication status	Organism Name	Origin / cultivar	Sex
SRR5627780	SRS2238707	cultivated	*Vitis vinifera*	Thompson RLK	Hermaphrodite
SRR5627781	SRS2238705	cultivated	*Vitis vinifera*	Muscat of Alexandria	Hermaphrodite
SRR5627782	SRS2238704	cultivated	*Vitis vinifera*	Thopson 2A	Hermaphrodite
SRR5627783	SRS2238703	wild	*Vitis vinifera subsp. sylvestris*	Turkmenistan1	Malqe
SRR5627784	SRS2238702	wild	*Vitis vinifera subsp. sylvestris*	Armenia	Female
SRR5627785	SRS2238701	wild	*Vitis vinifera subsp. sylvestris*	Pakistan1	Female
SRR5627786	SRS2238700	wild	*Vitis vinifera subsp. sylvestris*	Turkmenistan2	Male
SRR5627787	SRS2238699	wild	*Vitis vinifera subsp. sylvestris*	Azerbaijan1	Female
SRR5627789	SRS2238698	wild	*Vitis vinifera subsp. sylvestris*	Georgia	Female
SRR5627790	SRS2238696	wild	*Vitis vinifera subsp. sylvestris*	Azerbaijan2	Female
SRR5627791	SRS2238695	wild	*Vitis vinifera subsp. sylvestris*	Pakistan3	Female
SRR5627792	SRS2238694	wild	*Vitis vinifera subsp. sylvestris*	Pakistan2	Male
SRR5627793	SRS2238693	cultivated	*Vitis vinifera*	Semillion	Hermaphrodite
SRR5627794	SRS2238692	cultivated	*Vitis vinifera*	Riesling	Hermaphrodite
SRR5627795	SRS2238691	cultivated	*Vitis vinifera*	Cabernet Sauvignon	Hermaphrodite
SRR5627796	SRS2238690	cultivated	*Vitis vinifera*	Primitivo	Hermaphrodite
SRR5627797	SRS2238689	cultivated	*Vitis vinifera*	Pinot Noir	Hermaphrodite
SRR5627798	SRS2238688	cultivated	*Vitis vinifera*	Gamay Noir	Hermaphrodite
SRR5627799	SRS2238687	cultivated	*Vitis vinifera*	Chardonnay	Hermaphrodite
SRR5627800	SRS2238686	cultivated	*Vitis vinifera*	Aramon	Hermaphrodite
SRR5627801	SRS2238684	cultivated	*Vitis vinifera*	Zinfindel	Hermaphrodite
SRR5627802	SRS2238685	cultivated	*Vitis vinifera*	Traminer	Hermaphrodite

Supplementary Table 8: Statistics of mapping and SNP-calling of a public whole-genome resequencing dataset of the ncbi that was mapped to the *Vitis sylvestris* genome.

SRA run ID	Status	Number of reads	Proportion of mapped reads	Proportion of paired reads
SRR5627780	cultivated	70,284,983	0.989	0.975
SRR5627781	cultivated	76,803,804	0.991	0.98
SRR5627782	cultivated	80,937,324	0.992	0.981
SRR5627783	wild	80,232,477	0.986	0.974
SRR5627784	wild	35,879,048	0.954	0.92
SRR5627785	wild	105,896,803	0.991	0.978
SRR5627786	wild	80,948,588	0.99	0.979
SRR5627787	wild	76,824,067	0.985	0.97
SRR5627789	wild	80,107,055	0.989	0.977
SRR5627790	wild	79,079,895	0.965	0.938
SRR5627791	wild	105,783,610	0.991	0.979
SRR5627792	wild	115,956,970	0.991	0.976
SRR5627793	cultivated	77,221,117	0.992	0.98
SRR5627794	cultivated	77,644,996	0.992	0.98
SRR5627795	cultivated	71,775,105	0.992	0.98
SRR5627796	cultivated	169,763,362	0.972	0.942
SRR5627797	cultivated	90,757,557	0.993	0.981
SRR5627798	cultivated	66,283,833	0.992	0.983
SRR5627799	cultivated	454,053,065	0.982	0.956
SRR5627800	cultivated	96,530,135	0.992	0.98
SRR5627801	cultivated	223,282,216	0.992	0.978
SRR5627802	cultivated	60,767,595	0.99	0.974

Supplementary Table 9: Summary of the genotype of 13 cultivars in the sex locus on chromosome 2 inferred from whole-genome resequencing data. Cultivars were genotyped at XY SNPs. Yh refers to the modified Y haplotypes in hermaphrodites. Raw SNP data are shown in Figure 1a.

SRA run ID	Origin / cultivar	Genotype 4.810-4.903 Mb	Genotype 4.903-4.21Mb
SRR5627780	Thompson RLK	XYh	XYh
SRR5627781	Muscat of Alexandria	XYh	XYh
SRR5627782	Thopson 2A	XYh	XYh
SRR5627793	Semillion	XYh	XYh
SRR5627794	Riesling	YhYh	XYh
SRR5627795	Cabernet Sauvignon	XYh	XYh
SRR5627796	Primitivo	XYh	XYh
SRR5627797	Pinot Noir	XYh	XX
SRR5627798	Gamay Noir	XYh	XX
SRR5627799	Chardonnay	YhYh	XYh
SRR5627800	Aramon	XYh	XX
SRR5627801	Zinfindel	XYh	XYh
SRR5627802	Traminer	XYh	XX

Supplementary Table 10: Predicted genes in the sex locus of *V. sylvestris*. Coordinates and geneID are indicated for the *V. sylvestris* reference genome, therefore the absolute position of X-deleted genes is not shown.

Gene ID	Start	End	Strand	Functional annotation	Genotype in hermaphrodites
**000003Fg0028711**	4,810,035	4,815,364	-	trehalose phosphate phosphatase (TPP)	from exon 9, XYh or YhYh
**000003Fg0028721**	4,825,791	4,826,183	-	Protein INAPERTURATE POLLEN1 (INP1)	XYh or YhYh
**000003Fg0028731**	4,833,463	4,834,804	+	xyloglucan galactosyltransferase (GT19)	XYh or YhYh
**000003Fg0028741**	4,835,150	4,846,883	-	3-oxoacyl-[acyl-carrier-protein] synthase (KASIII)	XYh or YhYh
**NA**				Ethylene-overproducer-like1 / No Pollen Germination 1 (ETOL1)	XYh or YhYh
**000003Fg0028751**	4,861,687	4,862,981	+	transcription of the PLATZ family (VviPLATZ)	XYh or YhYh
**000003Fg0028761**	4,864,219	4,864,291	+	tRNA-gly	XYh or YhYh
**000003Fg0028771**	4,864,636	4,868,462	-	flavin mono-oxygenase (FMO)	XYh or YhYh
**000003Fg0028781**	4,870,175	4,873,359	-	flavin mono-oxygenase (FMO)	XYh or YhYh
**NA**				ATP-dependent DEAD BOX helicase (UAP56)	XYh or YhYh
**NA**				NUCLEOSOME ASSEMBLY PROTEIN 1 (NAP1)	XYh or YhYh
**NA**				Uncharacterized protein (UNK)	
**000003Fg0028791**	4,875,086	4,878,202	-	flavin mono-oxygenase (FMO)	XYh or YhYh
**000003Fg0028801**	4,887,323	4,890,854	-	flavin mono-oxygenase (FMO)	XYh or YhYh
**000003Fg0028811**	4,894,375	4,899,225	+	Uncharacterized protein (VviFSEX)	XYh or YhYh
**000003Fg0028821**	4,901,701	4,903,864	+	WRKY transcription factor 21 (WRKY21)	XYh or YhYh
**000003Fg0028831**	4,915,923	4,921,889	+	adenine phospho-ribosyltransferase (APRT3)	XYh or YhYh

Supplementary Table 11: Mapping statistics of public RNA-seq libraries of flower buds of female, male and hermaphrodite *V. sylvestris* (Ramos et al. 2013) against the *V. sylvestris* genome. B to D represent successive developmental stages.

SRA ID	Sex	Stage	Number of reads	% of reads mapped	% of reads assigned to a gene
SRR1239522	Female	B	13,571,346	95	84.2
SRR1239524	Female	D	14,596,473	95.5	84.9
SRR1239526	Female	G	14,111,102	95.4	84.3
SRR1239528	Female	H	18,464,325	94.7	82.9
SRR1239530	Male	B	17,247,932	95	84.1
SRR1239532	Male	D	17,244,273	94.7	83.7
SRR1239534	Male	G	7,711,531	95.3	84.1
SRR1239536	Male	H	17,830,620	94.5	83.6
SRR1239537	Hermaphrodite	B	19,832,905	94.2	83.4
SRR1239539	Hermaphrodite	D	13,814,366	94.9	83.7
SRR1239541	Hermaphrodite	G	14,239,068	95	83.1
SRR1239543	Hermaphrodite	H	15,564,147	96	83.9

Supplementary Table 12: Public RNA-seq dataset of *V. vinifera* and *V. sylvestris* mapped to the *Vitis sylvestris* genome.

SRA ID	Description	Cultivar	Data
SRR1709065	BERRY2.WD1	Pinot Noir	Illumina Single
SRR1709064	BERRY2.WD2	Pinot Noir	Illumina Single
SRR1709063	BERRY2.WD3	Pinot Noir	Illumina Single
SRR1709068	BERRY2.WW1	Pinot Noir	Illumina Single
SRR1709067	BERRY2.WW2	Pinot Noir	Illumina Single
SRR1709066	BERRY2.WW3	Pinot Noir	Illumina Single
SRR5989171	BERRY3.EL35.S1	Riesling	Illumina Single
SRR5989170	BERRY3.EL35.S2	Riesling	Illumina Single
SRR5989174	BERRY3.EL36.S1	Riesling	Illumina Single
SRR5989175	BERRY3.EL36.S2	Riesling	Illumina Single
SRR5989178	BERRY3.EL38.S1	Riesling	Illumina Single
SRR5989177	BERRY3.EL38.S2	Riesling	Illumina Single
SRR3056887	BUD2.Efs1	Prieto Picudo	Illumina Paired
SRR3056888	BUD2.Efs2	Prieto Picudo	Illumina Paired
SRR3056889	BUD2.Efs3	Prieto Picudo	Illumina Paired
SRR3056884	BUD2.Nfs1	Prieto Picudo	Illumina Paired
SRR3056885	BUD2.Nfs1	Prieto Picudo	Illumina Paired
SRR3056886	BUD2.Nfs1	Prieto Picudo	Illumina Paired
SRR2043187	BUD3.PC1	Corinto Bianco	Illumina Paired
SRR2043218	BUD3.PC2	Corinto Bianco	Illumina Paired
SRR2043221	BUD3.PC3	Corinto Bianco	Illumina Paired
SRR2043222	BUD3.SD1	Pedro Ximenes	Illumina Paired
SRR2043223	BUD3.SD2	Pedro Ximenes	Illumina Paired
SRR2043224	BUD3.SD3	Pedro Ximenes	Illumina Paired
SRR1553992	FLO.CK.1h.12DBB	Labruscan grape	Illumina Single
SRR1553993	FLO.CK.1h.12DBB	Labruscan grape	Illumina Single
SRR1554086	FLO.CK.24h.11DBB1	Labruscan grape	Illumina Single
SRR1554085	FLO.CK.24h.11DBB2	Labruscan grape	Illumina Single
SRR1553990	FLO.GA3.1h.12DBB	Labruscan grape	Illumina Single
SRR1553991	FLO.GA3.1h.12DBB	Labruscan grape	Illumina Single
SRR1553996	FLO.GA3.24h.11DBB1	Labruscan grape	Illumina Single
SRR1554080	FLO.GA3.24h.11DBB2	Labruscan grape	Illumina Single
SRR1302044	FLO2.SDL	Not specified	Illumina Paired

SRR1302041	FLO2.WT	Not specified	Illumina Paired
SRR519455	LEAF1.1DPI	Pinot Noir	Illumina Paired
SRR519456	LEAF1.2DPI	Pinot Noir	Illumina Paired
SRR519449	LEAF1.ctl1	Pinot Noir	Illumina Paired
SRR519450	LEAF1.ctl2	Pinot Noir	Illumina Paired
SRR519452	LEAF1.ctl3	Pinot Noir	Illumina Paired
SRR6706487	LEAF2.4DD1	Pinot Noir	Illumina Paired
SRR6706486	LEAF2.4DD2	Pinot Noir	Illumina Paired
SRR3056876	LEAF4.MtLS	Chasselat Cioutat	Illumina Paired
SRR3056875	LEAF4.MtLS	Chasselat Cioutat	Illumina Paired
SRR1926309	Mix.Wild.Ctl.1DPI1	Wild	Illumina Single
SRR1926307	Mix.Wild.Ctl.1DPI2	Wild	Illumina Single
SRR1926305	Mix.Wild.Ctl.1DPI3	Wild	Illumina Single
SRR1926310	Mix.Wild.Ctl.5DPI1	Wild	Illumina Single
SRR1926308	Mix.Wild.Ctl.5DPI2	Wild	Illumina Single
SRR1926306	Mix.Wild.Ctl.5DPI3	Wild	Illumina Single
SRR1926303	Mix.Wild.In.1DPI1	Wild	Illumina Single
SRR1926300	Mix.Wild.In.2DPI1	Wild	Illumina Single
SRR1926299	Mix.Wild.In.3DPI1	Wild	Illumina Single
SRR1926304	Mix.Wild.In.5DPI1	Wild	Illumina Single
SRR1926302	Mix.Wild.In.5DPI2	Wild	Illumina Single
SRR1926301	Mix.Wild.In.5DPI3	Wild	Illumina Single
SRR6300212	SEED.noSD.1	Red Globe X Crimson Seedless F1 hybrid	Illumina Paired
SRR6300211	SEED.noSD.2	Red Globe X Crimson Seedless F1 hybrid	Illumina Paired
SRR6300209	SEED.SD.1	Red Globe X Crimson Seedless F1 hybrid	Illumina Paired
SRR6300208	SEED.SD.2	Red Globe X Crimson Seedless F1 hybrid	Illumina Paired
DRR093297	STEM1.1	Pinot Noir	Illumina Single
DRR093298	STEM1.2	Pinot Noir	Illumina Single

Supplementary Text

Results of BAC sequencing and assembly

The final results were: 1) a female haplotype composed of the overlapping of 08J18 and 20H04 BAC sequences, of a total 204,082 bp length. This haplotype maps correctly between position 4,866,319 and 5,119,669 of the chromosome 2 of the *Vitis vinifera* reference genome (cultivar PN40024, 12X.V0, NCBI), which is known to correspond to the female haplotype of the Pinot Noir cultivar (Fechter et al 2012, Picq et al 2014); and 2) a male haplotype composed of two non-overlapping contigs, contig1: BACs 27H5 and 65K18 (171,096bp) and contig2: BACs 9D16, 15F19 and 71N3 (169,751bp). The Y haplotype does not map well on the grape genome reference, as the Yh haplotype of the PN40024 was not assembled on the chromosome 2 but its differing sequences were left unassigned (chromosome unknown). On the reference genome 12X.V0, the borders of the contig1 map at positions chr2:4,856,490 and chrUn:16,079,507; the borders of contig2 map at chr2:4,953,113 and chr:5,142,793. Based on the closest blast hits of the male contigs on the X and Yh haplotypes of the reference genome, as well as on the other available long-reads PacBio genomes (the Chardonnay sequences of Zhou et al 2019[19]; the Cabernet Sauvignon sequences of Chin et al 2016[9]; as well as the *Vitis sylvestris* female sequences of this work), we estimated the length of the gap between contig1 and contig2 at around 13kb.

Covering the 13kb gap on the Y haplotype

Further efforts were attempted to cover the gap between the two non-overlapping male contigs. As a gap of 13kb is too long to be covered by long-range PCR even when using high-quality TAQ, we made the hypothesis that the *PLATZ* gene, for which we have positional evidence in several female and hermaphrodite genotypes, is positioned within this gap in the Y haplotype too, and can be used to split the 13kb in two shorter parts, easier to amplify by long-range PCR. The positions of *PLATZ* on Yh and X haplotypes were obtained from: the reference genome of PN40024, on which the *PLATZ* female allele maps at chr2:4949289..4950582 and its hermaphrodite allele at chrUn16091133..16092418; the Chardonnay PacBio sequences (Zhou et al 2019); the Cabernet Sauvignon PacBio sequences (Chin et al 2016); as well as the *Vitis sylvestris* female PacBio sequences (this work). In addition, the *PLATZ* male allele was also found by microassembly of reads carrying Y alleles detecting in this work from the males of the *Vitis sylvestris* cross (this work); for facility we call this male version *PLATZy*.

Based on this hypothetical construct (contig1-gap1-PLATZy-gap2-contig2), we defined several pairs of primers based on the contig1- and contig2 ends and on *PLATZy*. The primers were defined exploiting polymorphisms between the X and Y haplotype, so to be sure to amplify only the Y haplotype. Starting from primers based on *PLATZy* and contig2 (forward: ACTCCCCTGTTTCTCTCCGA and reverse: TCATGTTGCGTCTAGATCGGT), we were able to obtain a single band of 3.2kb, corresponding to gap2, by long-range PCR on the *Vitis sylvestris* PSL10 male. DNAs from one female *V. sylvestris* and the hermaphrodite Pinot noir

(respectively with introduction codes 8500Mtp110 and 193Mtp81, INRA Vassal Grape Collection) did not provide any amplification, as expected. The forward and reverse Sanger sequences of this PCR product were obtained through an AB1 sequencer, and have 93-99% identity with the same region on 3' of *PLATZ*, of the reference genome PN40024, and the Cabernet Sauvignon, the Chardonnay and the *Vitis sylvestris* female PacBio sequences. We can thus confirm that in the Y haplotype, *PLATZy* is located in the expected region between *ETOL1* and the first *FMO*.

On the other hand, we did not succeed to sequence gap1, as PCR amplifications using several combinations of primers defined in contig1 and *PLATZ* always provided multiple bands, confirming that gap1 is highly likely to correspond to a repeated element, as observed in the reference genome PN40024, Cabernet Sauvignon, Chardonnay and *Vitis sylvestris* female.
