# Supplementary material for: The wild grape genome sequence provides insights into the transition from dioecy to hermaphroditism during grape domestication
Source: Genome Biol. 2020 Sep 7;21:223. doi: 10.1186/s13059-020-02131-y (PMC7487632; doi:10.1186/s13059-020-02131-y)
Supplement: Supplementary file 7 — Additional file 7. Review History. [file 13059_2020_2131_MOESM7_ESM.docx]

**Review history**

**First round of review**

**Reviewer 1**

The manuscript by Badouin, Velt and colleagues describes the identification and characterisation of candidate genes for sex determinism in wild and domesticated grapevine. A new genome assembly for Vitis sylvestris was developed, re-sequencing of a V sylvestris family and public sequences was used to identify variants present in the various haplotypes (male, female and hermaphrodite) and a list of candidates genes that are present in the locus and differentially expressed has been proposed. The manuscript does not present any functional characterisation of these candidate genes and their variants. The manuscript is well written and the order of experiments presented is logical. The analysis were done correctly and the conclusions are supported by the data. I only have a few minor comments.

My main concern is about the organisation of the manuscript. A few sections in Results have some content that belongs to the Discussion and not Results. This includes lines 156-157, 219-226, 231-232, 235-244, 269-271, 275-278 and 280-286.

Line 109: rephrase the first sentence to "We produced a de novo genome assembly for V. sylvestris. " Indicate the data obtained was from PacBio.

Lines 122-123: rephrase the sentence to "To precisely identify the boundaries of the sex locus, a F1 family obtained from a controlled cross of V. sylvestris was generated and re-sequenced. Paired-end Illumina short reads of both parents and 10 offspring yielded 43 to 74 millions of paired-end reads per individuals ()".

Line 144: remove "also"
Line 264: what does "consistent" mean? This is too subjective and some quantified differences between this new genome and those of Zinfandel, Chardonnay and Cabernet Sauvignon should be given.
Line 237: "sativa" instead of "vinifera".
Line 282: what is the right spelling? ViviSEX or VviFSEX? There is inconsistent spelling in the manuscript.
Line 296: 1.7Mb
Lines 362 to 369: can the age of TE be estimated?
Line 383: haplotypes
Page 28: capitalise BLATP, NCBI and Circos
Line 978: "marks" instead of "traits"
Line 1002: "… but it has not been sequenced yet."
Line 1005: the DOG protein domain is not mentioned in the main text of the manuscript.

**Reviewer 2**

The ms by Badouin et al. carries out genome sequencing and assembly of the wild progenitor of grapevine (V. vinifera), V. Sylvestris, in order to compare  the sex chromosomes of the dioecious Vitis sylvestris with the homologous Yh chromosome of the cultivated hermaphroditic species. The authors characterise the Y differentiated region containing the sex locus and identify candidate genes with crucial role in the development of male and female flower components.

The candidate genes described here for the suppression of gynoecium and androecium in male and female plants of the wild progenitor remain to be validated by experimental gene knock down. However, given the small size of the Y differentiated region (roughly 100 Kb), and the functional annotations and expression analyses provided by the authors, those genes (or some of them) remain the best candidates for having promoted dioecy in V. sylvestris.

This is a very interesting report which tries to get insights into the transition from dioecy to cosexuality during the domestication of a crop species which has enormous economic importance. For these reasons, I support publication on Genome Biology.

I have only few minor comments:
Line 299-300: 'we found that the sex locus.. is 111 Kb long'. (And throughout the ms). Please be aware that your analysis using XY SNPs defines the Y differentiated non-recombining region, NOT the sex determining region (i.e. the sex locus), which is included somewhere within the Y differentiated, albeit small, region. I understand that the position of your candidate genes for male and female sterility encompass most of the 111 Kb region, but since their role in determining dioecy remain to be ascertain, I would apply caution here.

Line 194-202. It is really puzzling that the transition from Y to Yh  occurred in the time scale of million of years, whereas the date for domestication of grapevine is in the range of thousands of years. How can you reconcile that? What is the generation time for grapevine, and is it different from that of V. sylvestris ?
Line 338-343. "However, more than two candidates were identified". You are suggesting that more genes might have been involved in producing dioecy. However, some of these deleted candidate genes might have become so, as a consequence of a secondary loss, because redundant and no longer important for female (or male) function.
Line 633: 'may be larger' change to 'may appear larger'
Line 641: 'the limits of the sex determining region' change to 'the boundaries of the Y differentiated region'.
Line 669: 'Whole haplotype comparisons were visualised with circos"? Is Circos a software? If so, please provide a reference.
Line 977: 'Red, purple, and blue, and white" , change to "Red, purple, blue and white"
Line 978: What do YY genotypes represent? From your figure 1, two grapevine cultivars have an extended region defined by the occurrence of YY genotypes.   Why this enrichment in these two cultivars? And why YY genotypes are found in the female plants of V. sylvestris?

**Reviewer 3**

Badouin et al generate a new genome assembly of wild grape and use this to study the structure of the region surrounding the sex-determining locus/loci. They use BAC sequencing to assembly the putative Y (male-specific) region and compare this to the regions of the X (female-specific) and Xh (hermaphrodite) haplotypes. The results suggest that the sex-determining region (X/Y haplotypes) are old and most likely ancestral in the genus. The Xh haplotype is substantially younger but the origin precedes the domestication of grape. Using a combination of presence/absence of genes on the X and Y haplotypes in combination with gene expression data during flower development they pinpoint a number of candidates genes for male and female sterility mutations.

The authors conclude that recombination across the sex-determining region appears to have ceased at once and there is no evidence of successive recruitment of genes to the regions. However, recombination suppression does not involve any of the usual mechanisms known to reduce recombination (e.g inversions). In light of this, the one thing I was missing from the study was data on any differences in epigenetic modifications, such as cytosine methylation, between the different haplotypes of the sex-determining region (X, Y, Yh). There is evidence for the accumulation of transposable elements on both the Y and Yh haplotypes, and since TEs are known to drive changes in methylation it is unfortunate that this information is not provided. Methylation is known to influence both the frequency of recombination and alter gene expression, so a lot of the observations the authors provide could possibly be linked to underlying differences in methylation between the different sex-determining haplotypes. Adding this type of data could really help narrow down the possible explanations for both the reduction/absence of recombination across the sex-determining region as well as the altered gene expression seen between X and Y copies of the homologous genes.

I think the calculations of the divergence times of the X and Y haplotypes (and also the Y and Yh haplotypes) could be clarified in more detail. It is never actually stated what mutation rate is used in these calculations, only a reference to Ossowski et al is given in the text. Also, the age calculations do include uncertainties in the generation time of Vitis, but does not indicate if uncertainties in the mutation rates were also considered.

The divergence between homologous genes on the X and Y haplotypes average dS~0.03. How does that compare to divergence among different species in the Vitis genus? If the divergence of the X and Y haplotypes predate the radiation of the entire genus, most species should have dS values <0.03 and that seems very low to me. Is there data available of dS values across multiple species of Vitis that can be cited to support this?

The legend for Figure 4 appears to be incomplete. There is only a reference for the a) panel and the remaining panels are not described at al. In particular, panels e) and f) which summarise the data on male sterility genes are not even mentioned in the legend.

Minor comments:  

Line 147: Please explain in more detail in the text that the 13kb gap in the Y haplotype is in fact predicted to contain the PLATZy gene. This is spelt out in detail in the Supplementary materials, but it would be good to also briefly summarise this here too and it is was not immediately clear to me whether the 13kb gap represented sequences that were completely missing from the Y haplotype or not. This becomes apparent later in the main text, but it would be good to state this already here whet the 13kb gap is first discussed.

Lines 243-244: "We could not…." This line is awkward and there's likely a word or two that are missing. Please rephrase.

Lines 316: The generation time should be in years not million years, right?

**Authors’ response to reviewers**

Reviewer #1: The manuscript by Badouin, Velt and colleagues describes the

identification and characterisation of candidate genes for sex determinism in wild and

domesticated grapevine. A new genome assembly for Vitis sylvestris was developed, resequencing

of a V sylvestris family and public sequences was used to identify variants

present in the various haplotypes (male, female and hermaphrodite) and a list of

candidates genes that are present in the locus and differentially expressed has been

proposed. The manuscript does not present any functional characterisation of these

candidate genes and their variants. The manuscript is well written and the order of

experiments presented is logical. The analysis were done correctly and the conclusions

are supported by the data. I only have a few minor comments.

We thank Referee 1 for the positive feedback. We acknowledge the absence of

genetic/functional validation of our candidates. Transformation of grapevine is indeed a

tedious procedure. Therefore, genetic transformation has mostly been used to modify

relatively simple traits in grapevine, by overexpressing selected genes. To our

knowledge, genetic transformation of grapevine has never been used to specifically

modify a character as complex as flower development. CRISPR/Cas9-mediated targeted

mutagenesis is a promising tool for functional validation. However, application of this

approach to grapevine is still under development, with less than ten reports in PubMed

(as of May 2020). Finally, to our knowledge, no TILLING platform is available in Vitis.

Alternatively, model plant species such as Arabidopsis may be used for indirect

functional validation, but this strategy would nevertheless involve complex

transformation approaches, including heterologous complementation of Arabidopsis

flower mutants. Altogether, we believe that the functional validation of any of the

candidate genes potentially involved in sex determination in grapevine is a medium-term

project (about 3 years) in itself and should be part of another manuscript.

My main concern is about the organisation of the manuscript. A few sections in Results

have some content that belongs to the Discussion and not Results. This includes lines

156-157, 219-226, 231-232, 235-244, 269-271, 275-278 and 280-286.

156-157: we added an equivalent sentence in Discussion, section “Molecular evolution

of the grapevine sex locus” lines 296-297.

219-226, 231-232, 235-244, 269-271, 275-278 and part of 280-286 were moved to a

new section of the Discussion called “A genetic model for the sex determination of wild

and cultivated grapevine” lines 303-338. We also moved lines 351-354 from “Sex

determination in wild and cultivated grapevine” to this new section to conclude it.

The remainder of 280-286 was moved to Discussion section formerly called “Sex

determination in wild and cultivated grapevine”, and now called “Evolution of sex

determination in plants: insights from grapevine” line 372.

We have further re-structured Discussion and the end of the former section “Sex

determination in wild and cultivated grapevine” now forms a new section called

“Changes in the sex locus and grapevine domestication”.

Line 109: rephrase the first sentence to "We produced a de novo genome assembly for

V. sylvestris. " Indicate the data obtained was from PacBio.

Done.

Lines 122-123: rephrase the sentence to "To precisely identify the boundaries of the sex

locus, a F1 family obtained from a controlled cross of V. sylvestris was generated and

re-sequenced. Paired-end Illumina short reads of both parents and 10 offspring yielded

43 to 74 millions of paired-end reads per individuals ()".

Done.

Line 144: remove "also"

Done.

Line 164: what does "consistent" mean? This is too subjective and some quantified

differences between this new genome and those of Zinfandel, Chardonnay and

Cabernet Sauvignon should be given.

We now give some details. The comparison between our Cabernet Sauvignon H

haplotype and the published one showed very high similarity (99.97%), the differences

were found in repeats (TTTT vs TTT for example). The comparison between our

Cabernet Sauvignon H haplotype and the Chardonnay H’ haplotype returns 99.62% of

identity, and that between our Cabernet Sauvignon H haplotype and the Zinfandel H

haplotype, 99.93%. Gene content and gene order were the same as shown by the dot

plots below. These were not included in the supplementary material as it is already very

large, but if the referee and the editor think if it is necessary to include them, we will, of

course.

Line 237: "sativa" instead of "vinifera".

Done.

Line 282: what is the right spelling? ViviSEX or VviFSEX? There is inconsistent spelling

in the manuscript.

The right spelling is VviFSEX. This has been corrected. We made further gene name

spelling corrections in Figure 4 and 5.

Line 296: 1.7Mb

Done.

Lines 362 to 369: can the age of TE be estimated?

As far as we know, two approaches are possible: estimating the divergence of the LTRs

of the same copy, or computing the divergence of all the copies of an element from the

consensus and get a distribution. First, it is difficult to translate these estimates in

millions years, and it is not clear how we would compare them to the estimates we got

from genes. Moreover, the presence of TEs specific to Yh (not found in X or Y) clearly

indicates that they accumulated during or after domestication, which is our main point

here.

Line 383: haplotypes

Done.

Page 28: capitalise BLATP, NCBI and Circos

Done.

Line 978: "marks" instead of "traits"

Done.

Line 1002: "… but it has not been sequenced yet."

Done.

Line 1005: the DOG protein domain is not mentioned in the main text of the manuscript.

We now mention it in the main text when introducing INP1.

Reviewer #2: The ms by Badouin et al. carries out genome sequencing and assembly of

the wild progenitor of grapevine (V. vinifera), V. Sylvestris, in order to compare the sex

chromosomes of the dioecious Vitis sylvestris with the homologous Yh chromosome of

the cultivated hermaphroditic species. The authors characterise the Y differentiated

region containing the sex locus and identify candidate genes with crucial role in the

development of male and female flower components.

The candidate genes described here for the suppression of gynoecium and androecium

in male and female plants of the wild progenitor remain to be validated by experimental

gene knock down. However, given the small size of the Y differentiated region (roughly

100 Kb), and the functional annotations and expression analyses provided by the

authors, those genes (or some of them) remain the best candidates for having promoted

dioecy in V. sylvestris.

This is a very interesting report which tries to get insights into the transition from dioecy

to cosexuality during the domestication of a crop species which has enormous economic

importance. For these reasons, I support publication on Genome Biology.

We thank referee 2 for the positive feedback. We acknowledge the absence of

genetic/functional validation of our candidates. See our reply to the same comment by

referee 1.

I have only few minor comments:

Line 299-300: 'we found that the sex locus.. is 111 Kb long'. (And throughout the ms).

Please be aware that your analysis using XY SNPs defines the Y differentiated nonrecombining region, NOT the sex determining region (i.e. the sex locus), which is

included somewhere within the Y differentiated, albeit small, region. I understand that

the position of your candidate genes for male and female sterility encompass most of the

111 Kb region, but since their role in determining dioecy remain to be ascertain, I would

apply caution here.

We completely agree with the referee. However, this over simplification is made in the

literature about Vitis sex determination. We maintained it to avoid making the readers

confused. We included two sentences explaining this over-simplification lines 272-275.

Line 194-202. It is really puzzling that the transition from Y to Yh occurred in the time

scale of million of years, whereas the date for domestication of grapevine is in the range

of thousands of years. How can you reconcile that? What is the generation time for

grapevine, and is it different from that of V. sylvestris ?

We agree with referee 2 that grapevine domestication was in the range of thousands of

years. Our explanation is that domestication relied on standing variation. Yh was already

there when domestication started. Crypto-trioecy (male, female, with a very small

fraction of hermaphrodites) is very common in dioecious plants, including relatively old

dioecious species with differentiated sex chromosomes. Our age estimates of Y and Yh

are tentative as they rely on imprecise molecular clocks. We need some data to confirm

them (for example experimentally measured mutation rate in grapevine, phylogenetic

analysis of X, Y and Yh over many cultivars, etc…), which we hope other studies will

bring.

Line 338-343. "However, more than two candidates were identified". You are suggesting

that more genes might have been involved in producing dioecy. However, some of these

deleted candidate genes might have become so, as a consequence of a secondary loss,

because redundant and no longer important for female (or male) function.

This is a good point. We changed the discussion. It is difficult to conclude solely with our

data. However, data on several Vitis from another group tend to confirm this view.

Line 633: 'may be larger' change to 'may appear larger'

Done.

Line 641: 'the limits of the sex determining region' change to 'the boundaries of the Y

differentiated region'.

Done.

Line 669: 'Whole haplotype comparisons were visualised with circos"? Is Circos a

software? If so, please provide a reference.

Done.

Line 977: 'Red, purple, and blue, and white" , change to "Red, purple, blue and white"

Done.

Line 978: What do YY genotypes represent? From your figure 1, two grapevine cultivars

have an extended region defined by the occurrence of YY genotypes. Why this

enrichment in these two cultivars? And why YY genotypes are found in the female plants

of V. sylvestris?

This analysis shows the status of the X-Y SNPs found in our cross in cultivated and wild

grapevine found in public databases. YY means that both Y alleles were found at a SNP.

It is well known that some cultivars have two Yh haplotypes, most Y alleles are shared

between Y and Yh, hence the results for the two cultivars pointed by referee 2. These

cultivars are Chardonnay and Riesling (see supplementary table 9). There data are a bit

noisy (some allelic combination are found in the wrong sex, e.g. YY in females), but the

overall signal is very clear. We changed figure 1 legend to be clearer.

Reviewer #3: Badouin et al generate a new genome assembly of wild grape and use this

to study the structure of the region surrounding the sex-determining locus/loci. They use

BAC sequencing to assembly the putative Y (male-specific) region and compare this to

the regions of the X (female-specific) and Xh (hermaphrodite) haplotypes. The results

suggest that the sex-determining region (X/Y haplotypes) are old and most likely

ancestral in the genus. The Yh haplotype is substantially younger but the origin

precedes the domestication of grape. Using a combination of presence/absence of

genes on the X and Y haplotypes in combination with gene expression data during

flower development they pinpoint a number of candidates genes for male and female

sterility mutations.

The authors conclude that recombination across the sex-determining region appears to

have ceased at once and there is no evidence of successive recruitment of genes to the

regions. However, recombination suppression does not involve any of the usual

mechanisms known to reduce recombination (e.g inversions). In light of this, the one

thing I was missing from the study was data on any differences in epigenetic

modifications, such as cytosine methylation, between the different haplotypes of the sexdetermining

region (X, Y, Yh). There is evidence for the accumulation of transposable

elements on both the Y and Yh haplotypes, and since TEs are known to drive changes

in methylation it is unfortunate that this information is not provided. Methylation is known

to influence both the frequency of recombination and alter gene expression, so a lot of

the observations the authors provide could possibly be linked to underlying differences

in methylation between the different sex-determining haplotypes. Adding this type of

data could really help narrow down the possible explanations for both the

reduction/absence of recombination across the sex-determining region as well as the

altered gene expression seen between X and Y copies of the homologous genes.

We agree with referee 3 that a study on the epigenetic marks in the X, Y and Yh

haplotypes would be really interesting. However, we do not have this data at hand. The

manuscript already includes a lot of data and results, listed by referee 3 above. We feel

that studying the epigenetics of the sex-determining region is beyond this work and

should be the focus of another manuscript.

I think the calculations of the divergence times of the X and Y haplotypes (and also the Y

and Yh haplotypes) could be clarified in more detail. It is never actually stated what

mutation rate is used in these calculations, only a reference to Ossowski et al is given in

the text. Also, the age calculations do include uncertainties in the generation time of

Vitis, but does not indicate if uncertainties in the mutation rates were also considered.

The actual rate is now indicated. It is the rate proposed by the authors (see abstract of

Ossowski et al. 2010). We already mentioned in the text that only “rough” estimates can

be obtained from this (see lines 183-185).

The divergence between homologous genes on the X and Y haplotypes average

dS~0.03. How does that compare to divergence among different species in the Vitis

genus? If the divergence of the X and Y haplotypes predate the radiation of the entire

genus, most species should have dS values <0.03 and that seems very low to me. Is

there data available of dS values across multiple species of Vitis that can be cited to

support this?

Comparing X and Y sequences to estimate the age of the sex chromosomes is a rather

standard procedure (see Charlesworth et al. 2005; Bergero & Charlesworth 2009; Muyle

et al. 2017). We nevertheless agree with referee 3 that comparing our dS estimate for

the sex locus with dS from pairs of Vitis species would be interesting. However, this

requires a clean set of orthologous genes. We identified such a dataset in Ma et al. 2018

(already cited in the ms for the phylogeny of the Vitis genus). Only the raw reads can be

downloaded for this paper. We contacted the authors but were unable to get alignments,

nor the CDS of their set of orthologous genes. Obtaining such a dataset requires a

significant amount of work, which is beyond the scope of this manuscript. The vitis

genus is known to evolve very slowly and the low dS value we got for the sex locus is

consistent with this observation, which is now stated in the Discussion, lines 280-282.

Moreover, our age estimate of the sex locus can be compared with that of Massonnet et

al. (2020) obtained from a completely different approach and both coincide, as already

stated.

The legend for Figure 4 appears to be incomplete. There is only a reference for the a)

panel and the remaining panels are not described at all. In particular, panels e) and f)

which summarise the data on male sterility genes are not even mentioned in the legend.

We thank the referee for spotting this omission. The legend was completed.

Minor comments:

Line 147: Please explain in more detail in the text that the 13kb gap in the Y haplotype is

in fact predicted to contain the PLATZy gene. This is spelt out in detail in the

Supplementary materials, but it would be good to also briefly summarise this here too

and it is was not immediately clear to me whether the 13kb gap represented sequences

that were completely missing from the Y haplotype or not. This becomes apparent later

in the main text, but it would be good to state this already here whet the 13kb gap is first

discussed.

Done.

Lines 243-244: "We could not…." This line is awkward and there's likely a word or two

that are missing. Please rephrase.

Done.

Lines 316: The generation time should be in years not million years, right?

Corrected. Thanks.

**Second round of review**

**Reviewer 1**

I am happy with the revisions.

**Reviewer 3**

The authors have addressed all the issues has with the first version of the ms. I have no further comments at this point.
